# SAV1 in Cooperation With FKBP52 Participates in Implantation

**DOI:** 10.1002/advs.77761

**Published:** 2026-09-16

**Authors:** Yuhan Shao, Yafang Lu, Zhaoyu Jia, Junxiang Ren, Ziyi Ge, Xinyu Liu, Hui Zhao, Zi‐Jiang Chen, Junhao Yan, Wei Wang, Jia Yuan

**Affiliations:** ^1^ State Key Laboratory of Reproductive Medicine and Offspring Health Center for Reproductive Medicine Advanced Medical Research Institute Cheeloo College of Medicine Shandong University Jinan Shandong China; ^2^ National Research Center for Assisted Reproductive Technology and Reproductive Genetics Institute of Women Children and Reproductive Health Shandong University Jinan Shandong China; ^3^ Ministry of Education Key Laboratory of Reproductive Endocrinology (Shandong University) Jinan Shandong China; ^4^ Interventional Medicine Department The Second Qilu Hospital Shandong University Jinan Shandong China

**Keywords:** embryo implantation, female fertility, FKBP52, hippo signaling pathway, P_4_ resistance, SAV1, uterine receptivity

## Abstract

Endometrial receptivity is restricted to an implantation window, during which progesterone (P_4_) and estrogen (E_2_) orchestrate uterine remodeling to support blastocyst attachment. While E_2_ is indispensable for receptivity, the P_4_‐primed uterus undergoes a transitional phase marked by luminal epithelial remodeling, stromal proliferation, and glandular differentiation, establishing competence for E_2_ action. Precise coordination of P_4_‐progesterone receptor (PR) signaling with estrogenic cues is essential; upstream regulatory mechanisms remain unclear. We identify Salvador homolog 1 (SAV1), a scaffold of the Hippo pathway, as a critical regulator of this temporal coordination. Deletion of *Sav1* in endometrial cells impaired gland development, disrupted receptivity, and prevented implantation. SAV1 interacts with FKBP52), a progesterone receptor cochaperone, and promotes its mammalian STE20‐like kinase 1/2 (MST1/2)‐dependent phosphorylation, facilitating FK506‐binding protein 52 (FKBP52) nuclear localization and enhancing PR activation. Loss of SAV1 blunted progesterone signaling, delayed receptive phase, and disrupted competency for implantation. Hormonally induced reactivation after delayed implantation restored receptivity and implantation success in *Sav1*‐deficient mice. These findings identify the MST1/2‐SAV1‐FKBP52 axis as an integrator that couples Hippo signaling with steroid receptor activity. By ensuring the temporal precision of progesterone action, SAV1 orchestrates establishment of uterine receptivity and implantation, revealing a previously unrecognized role of noncanonical Hippo signaling in regulating P_4_‐dependent responses that safeguard reproductive success.

## Graphical abstract

The MST‐SAV1 complex modulates FKBP52 phosphorylation to fine‐tune PR activity, ensuring timely uterine transition to receptivity under estrogen signaling for implantation. Stromal SAV1 scaffolds MST1/2‐mediated FKBP52 phosphorylation via noncanonical Hippo to promote PR activity. SAV1 loss causes progesterone resistance, receptivity misalignment, and implantation failure.

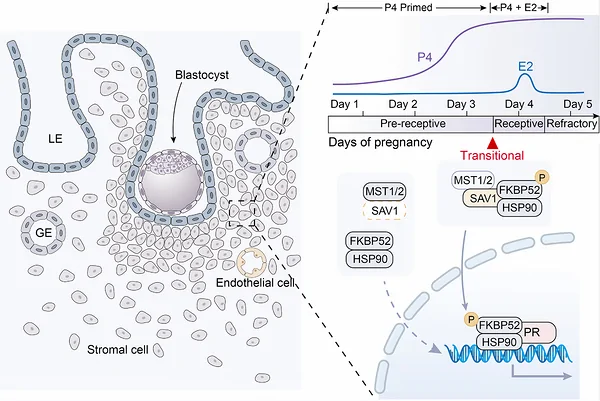

## 1 Introduction

Endometrial receptivity is confined to a transient “window of implantation” during which the uterine environment becomes conducive to blastocyst attachment and subsequent implantation [1, 2]. This tightly regulated state is orchestrated by the spatiotemporal interplay of ovarian progesterone (P_4_) and estrogen (E_2_). In mice, the uterus achieves receptive on day 4 of pregnancy (day 1 = vaginal plug) and subsequently transitions to the refractory state on day 5. Mouse models demonstrate that an aberrant hormonal signaling response balance between E_2_ and P_4_ in the uterus results in compromised receptivity and implantation defect [3, 4, 5]. Although E_2_ is indispensable for the uterine transition to receptivity, the P_4_‐primed uterus undergoes preparatory remodeling prior to E_2_ action through the regulated expansion and phenotypic specialization of distinct cellular populations [1, 6]. This process establishes the competence to enter the receptive endometrial state, known as the transitional phase [7], which is characterized by specific cellular events, such as luminal epithelial polarity remodeling, stromal cell proliferation, and glandular epithelial differentiation.

Progesterone exerts its biological effects primarily through the progesterone receptor (PR), which transcriptionally regulates target genes and activates pregnancy stage‐specific signaling pathways, particularly those required for uterine receptivity and implantation [8, 9]. The essential role of PR is underscored by the observation that blastocysts fail to implant in PR‐null mice [10]. PR activity is finely modulated by diverse mechanisms, including post‐translational modifications, epigenetic regulation, and interactions with co‐regulatory proteins. Dysregulation of PR function often manifests as P_4_ resistance, typified by aberrant uterine gene expression during the receptive phase, characterized by upregulation of E_2_‐responsive genes concomitant with downregulation of P_4_‐responsive genes, ultimately leading to implantation failure. Notably, progression to the transitional phase in a P_4_‐primed uterus requires precisely timed P_4_‐PR signaling, followed by coordination with estrogenic cues to establish the transient implantation window. However, the molecular basis of this temporal coordination remains to be elucidated.

The canonical Hippo signaling pathway is a highly conserved signal transduction cascade that plays a critical role in organ development and is broadly involved in biological processes such as carcinogenesis, tissue injury repair, and regeneration [11, 12, 13]. Mammalian STE20‐like kinase 1/2 (MST1/2) forms a complex with Salvador homolog 1 (SAV1), promoting the phosphorylation of large tumor suppressor kinase 1/2 (LATS1/2) by MST1/2, which in turn induces yes‐associated protein (YAP) phosphorylation, resulting in its retention in the cytoplasm [14, 15]. When SAV1 is absent, the Hippo pathway is disrupted, leading to YAP dephosphorylation and nuclear translocation, where it activates the transcription of downstream target genes, thereby regulating cell proliferation, differentiation, and tumorigenesis. Previous studies have demonstrated that the Hippo signaling pathway also plays a significant role in the female reproductive system, modulating follicle development and oocyte maturation [16]. In mouse uterine stromal cells, individual knockout of *Lats1* or *Lats2* does not impair female fertility, but dual knockout of *Lats1/2* results in Müllerian duct developmental abnormalities, leading to uterine malformations [17]. Recent studies have shown that *Yap1* and *Wwtr1* are required for decidualization [18, 19, 20]. Given that the Hippo pathway functions as a conserved signaling cascade, it remains unclear how upstream signals modulate its activity to influence specific events during pregnancy.

Our previous study indicated that peri‐implantation is associated with Hippo pathway activation [21], implicating a pivotal role for SAV1 in this process. To investigate the role of uterine *Sav1* in pregnancy events, we employed mouse models with uterus‐specific *Sav1* deletion using *Pgr‐Cre* or *Ltf‐Cre* drivers. Our findings demonstrate that *Sav1* deficiency in endometrial cells impairs uterine gland development, compromises receptivity, and hinders embryo implantation, whereas epithelial‐specific deletion of *Sav1* does not affect pregnancy outcomes. More importantly, we found that the SAV1‐MST complex regulates PR activity by modulating FK506‐binding protein 52 (FKBP52) phosphorylation, thereby orchestrating the timely progression of the uterus into the transitional phase, followed by entry into the receptive phase under synergistic estrogen action. This represents a novel function of the Hippo signaling via a non‐canonical pathway in regulating P_4_‐dependent responses in endometrial cells.

## 2 Results

### 2.1 Ablation of *Sav1* in the Mouse Uterus Leads to Severe Fertility Defects and Impairs Glandular Development

**FIGURE 1:**
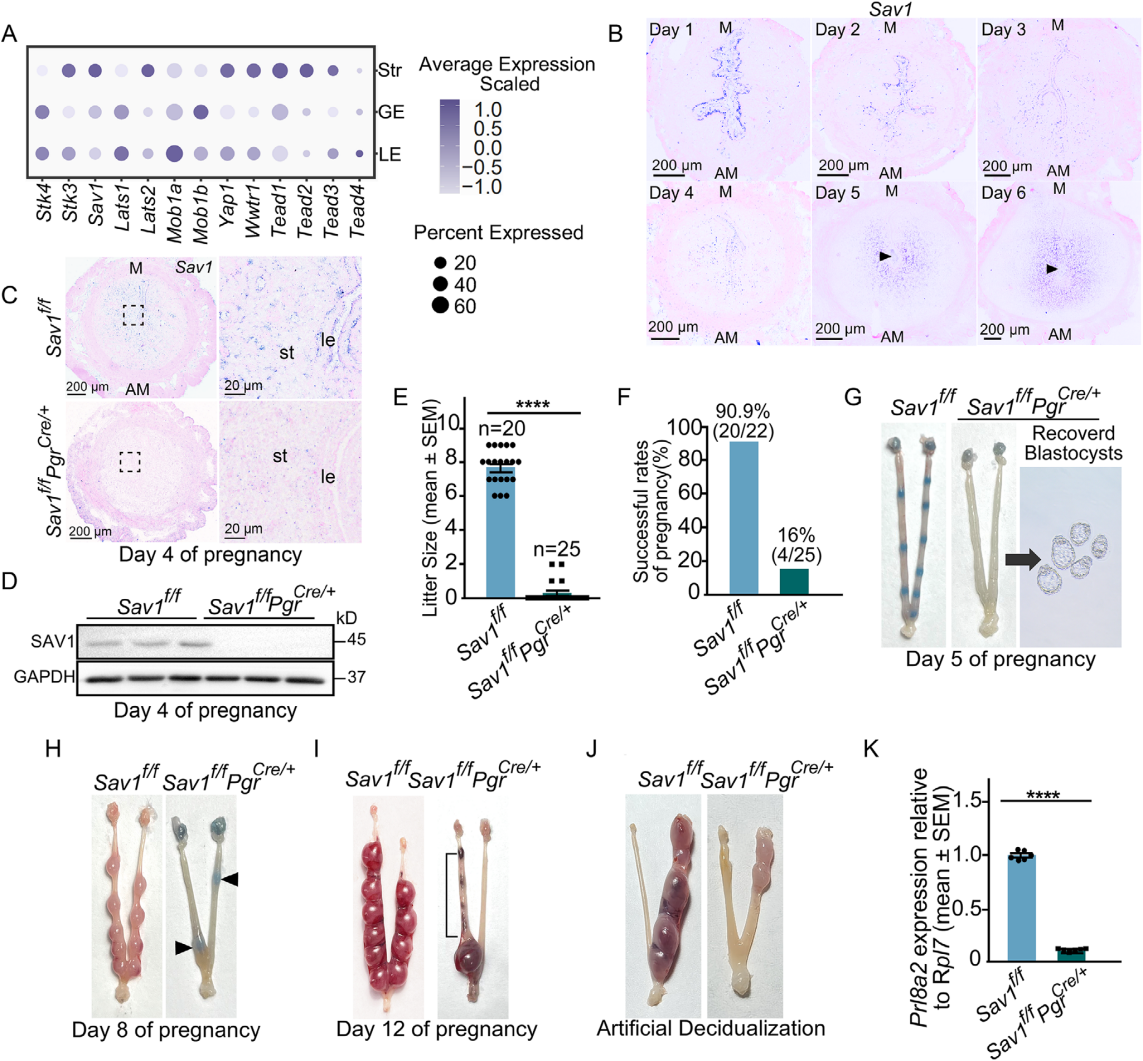
Uterine *Sav1* deletion severely impairs fertility due to implantation defect. (A) Dot plot showing genes expression of canonical Hippo pathway genes in LE (luminal epithelium), GE (glandular epithelium), and Str (stroma) clusters on day 4 of pregnancy. (B) In situ hybridization of *Sav1* mRNA in uteri from day 1–4 and implantation sites from day 5–6 of pregnancy. Arrowheads indicate embryos. Scale bars, 200 µm. (C) In situ hybridization of *Sav1* mRNA in day 4 *Sav1^f/f^* and *Sav1^f/f^Pgr^Cre/+^* uteri. Scale bars, 200 µm. Boxed areas are shown at higher magnification in the right panels. Scale bars, 20 µm. le, luminal epithelium; st, stroma. (D) Western blotting of SAV1 in uterine tissues from day 4 *Sav1^f/f^* and *Sav1^f/f^Pgr^Cre/+^* mice. GAPDH was used as a loading control. (E,F) Litter size and pregnancy success rates in *Sav1^f/f^* and *Sav1^f/f^Pgr^Cre/+^* females. Numbers in brackets indicate females giving birth/total plug‐positive females; n, number of females examined. (G) Implantation sites in *Sav1^f/f^* and *Sav1^f/f^Pgr^Cre/+^* females, and blastocysts recovered from *Sav1^f/f^Pgr^Cre/+^* uteri on day 5. The black arrow indicates the embryos (right panel) recovered from the corresponding uterus. (H) Day 8 implantation sites in *Sav1^f/f^* and *Sav1^f/f^Pgr^Cre/+^* females and arrowheads indicate weak and small blue bands. (I) Day 12 implantation sites in *Sav1^f/f^* and *Sav1^f/f^Pgr^Cre/+^* females. The bracket indicates adjacent resorption sites. (J) Decidual response of *Sav1^f/f^* and *Sav1^f/f^Pgr^Cre/+^* females. Right uterine horns were injected intraluminally with oil, and the left horn was left uninjected as a contralateral control. Only *Sav1^f/f^* mice displayed a marked increase in the size of the oil‐injected horn compared with the uninjected side. (K) qRT‐PCR analysis showing induced expression of the decidual gene *Prl8a2* in decidualized uterine tissues from *Sav1^f/f^* females (*n* = 6 mice per group). M, mesometrial pole; AM, antimesometrial pole. Statistical analysis was performed using Welch's *t*‐test. Data are presented as mean ± stand error of the mean (SEM). ^****^ *p* < 0.0001.

SAV1, a core component of the Hippo signaling, interacts with MST1/2 to enhance their kinase activity, thereby promoting phosphorylation of the LATS1/2‐MOB kinase activator 1A and 1B complex. Activated LATS1/2 subsequently phosphorylate and inactivate YAP and transcriptional coactivator with PDZ‐binding motif (TAZ), preventing TEA domain transcription factors 1‐4‐mediated transcription and executing the canonical Hippo kinase cascade [11, 13]. Our previous single‐cell sequencing data [7] revealed that key members of the canonical Hippo pathway are expressed in the mouse uterus on day 4 of pregnancy, with *Sav1* expressed in both stromal and epithelial cells (Figure 1A). To investigate the spatiotemporal expression of SAV1 during the peri‐implantation period, we performed in situ hybridization and found a dynamic transition of *Sav1* expression from the epithelium to a marked enrichment in decidual cells (Figures 1B and S1A).

To assess the function of *Sav1* during early pregnancy, we generated uterine‐specific *Sav1* knockout mice using a *Pgr‐Cre* driver (*Sav1^f/f^Pgr^Cre/+^*). Knockout efficiency was confirmed through in situ hybridization and Western blot (Figure 1C,D). *Sav1^f/f^Pgr^Cre/+^* females showed severe subfertility: Approximately 84% of plug‐positive females failed to produce any litters, and the remaining 16% of plug‐positive *Sav1^f/f^Pgr^Cre/+^* females produced significantly smaller litters (Figure 1E,F). To explore the cause of the subfertility, we assessed the success rate of blastocyst implantation on day 5. *Sav1^f/f^Pgr^Cre/+^* females displayed severe implantation defects, with morphologically normal blastocysts recovered floating in the uterine lumen (Figure 1G). Further examination of fertility phenotypes revealed that *Sav1^f/f^Pgr^Cre/+^* females exhibited markedly fewer and smaller implantation sites on day 8 compared with littermate floxed females (Figure 1H). By day 12, most conceptuses in *Sav1^f/f^Pgr^Cre/+^* females had undergone nearly complete resorption (Figure 1I). Furthermore, ablation of *Sav1* also impaired the decidual response with no uterine weight gain, morphological changes, or induction of *Prl8a2* expression following artificially induced decidualization (Figure 1J,K). In addition, serum hormone levels, luteal function, and uterine expression of estrogen receptor 1 (ESR1) and progesterone receptor (PGR) were not significantly altered in *Sav1^f/f^Pgr^Cre/+^* females (Figure S1B–F).

**FIGURE 2:**
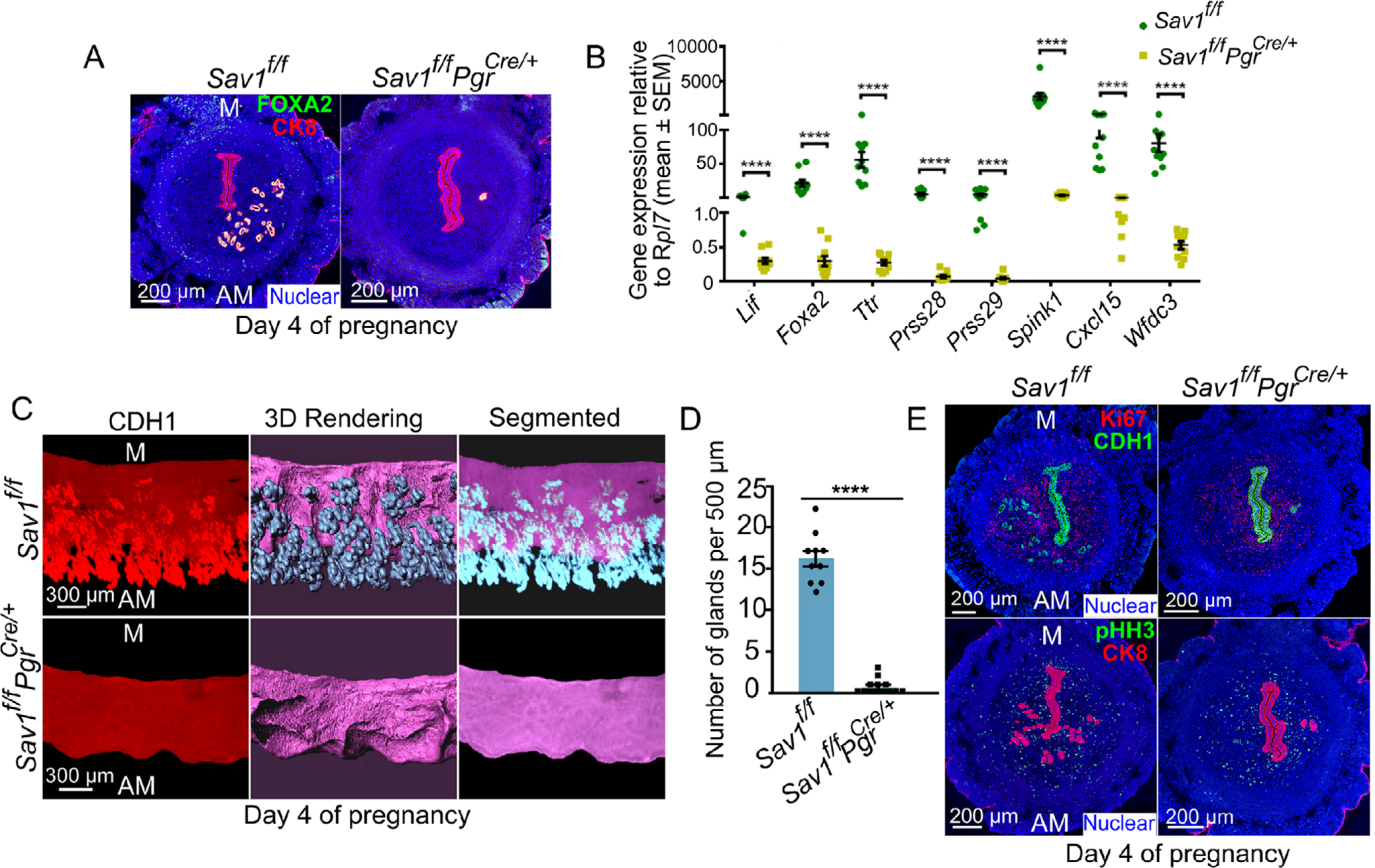
Defect of gland formation in *Sav1^f/f^Pgr^Cre/+^* uterus. (A) IF of FOXA2 and CK8 in *Sav1^f/f^* and *Sav1^f/f^Pgr^Cre/+^* uteri on day 4 of pregnancy. Scale bars, 200 µm. (B) qRT‐PCR analysis of glandular marker genes reveals severe defects in uterine gland development in *Sav1^f/f^Pgr^Cre/+^* mice on day 4 of pregnancy (*n* = 10 mice per group). (C) 3D imaging of day 4 pregnant uteri from *Sav1^f/f^* and *Sav1^f/f^Pgr^Cre/+^* females. Images of CDH1 immunostaining and 3D rendered images in each genotype show the deficient glandular structure in *Sav1^f/f^Pgr^Cre/+^* females. Luminal epithelium is shown in magenta, and glandular epithelium in blue. Images were generated by a Lightsheet whose Objective Detection was EC Plan‐Neofluar 5×/0.16 and illumination Objective was LSFM 5×/0.1. Scale bars, 300 µm. (D) Number of glands per 500 µm along the XZ‐axis orientation in 3D uteri counted from *Sav1^f/f^* (*n* = 10) and *Sav1^f/f^Pgr^Cre/+^* (*n* = 13) females utilizing the Imaris image analysis software. (E) IF of Ki67 and CDH1, pHH3 and CK8 in *Sav1^f/f^* and *Sav1^f/f^Pgr^Cre/+^* uteri on day 4 of pregnancy. Scale bars, 200 µm. M, mesometrial pole; AM, antimesometrial pole. Statistical analysis was performed using Welch's *t*‐test. Data are presented as mean ± SEM. ^****^ *p* < 0.0001.

Since glands are indispensable for embryo implantation [22, 23, 24], we utilized immunofluorescence (IF) staining for the epithelial marker cytokeratin 8 (CK8) and the glandular marker forkhead box 2 (FOXA2) in day 4 uterine sections. We found that *Sav1^f/f^Pgr^Cre/+^* day 4 uteri exhibited a substantially reduced number of glands compared with floxed mice (Figure 2A). Consistent with this, quantitative reverse transcription polymerase chain reaction (qRT‐PCR) showed significantly decreased levels of gland‐specific markers, including *Lif*, *Foxa2*, *Ttr*, *Prss28*, *Prss29*, *Spink1*, *Cxcl15*, and *Wfdc3* (Figure 2B). To visualize structural alterations in the uterine lumen‐gland architecture, we employed tissue clearing and captured three‐dimensional (3D) images of day 4 uteri, which confirmed defective gland formation in *Sav1^f/f^Pgr^Cre/+^* mice (Figure 2C), further quantified and validated by assessing gland units per uterine length (Figure 2D). Importantly, gland loss in *Sav1^f/f^Pgr^Cre/+^* mice cannot be attributed to abnormal proliferative dynamics of the endometrium in early pregnancy, as epithelial cells appropriately ceased proliferation and stromal cells proliferated actively on day 4 (Figure 2E). Taken together, these findings demonstrate that loss of *Sav1* leads to aberrant implantation and adverse pregnancy outcomes, with impaired gland development serving as a key contributing factor in *Sav1^f/f^Pgr^Cre/+^* mice.

### 2.2 Leukemia Inhibitory Factor Rescue Reveals Shifted “Window” of Implantation in Mice With Uterine *Sav1* Deletion

**FIGURE 3:**
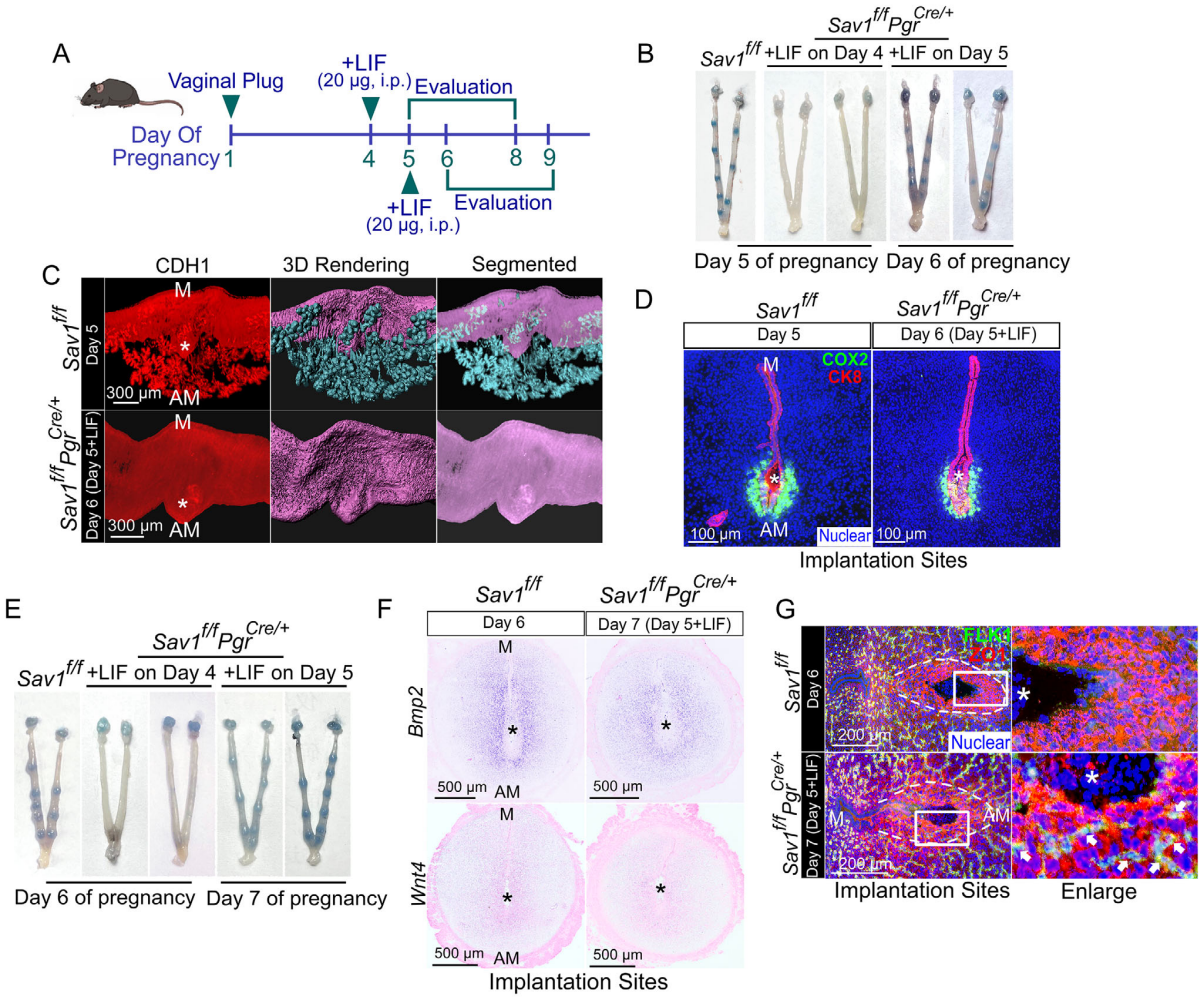
mLIF induction on day 5 of pregnancy in *Sav1^f/f^Pgr^Cre/+^* mice rescued implantation. (A) Schedule for recombinant mLIF administration and evaluation. (B) Representative photographs of uteri from *Sav1^f/f^* females on day 5 of pregnancy, and from *Sav1^f/f^Pgr^Cre/+^* females on day 5 and day 6 of pregnancy, following mLIF treatment on day 4 and day 5, respectively. (C) 3D visualization of implantation sites from *Sav1^f/f^* (day 5) and *Sav1^f/f^Pgr^Cre/+^* (day 6) females. CDH1 immunostaining images, along with 3D‐rendered and segmented reconstructions of day 6 implantation sites, reveal the implantation chamber in *Sav1^f/f^Pgr^Cre/+^* females that received mLIF injection on day 5 of pregnancy. Magenta denotes the luminal epithelium, whereas blue denotes the glandular epithelium. Scale bars, 300 µm. (D) IF of COX2 and CK8 in implantation sites from *Sav1^f/f^* females on day 5 of pregnancy, and from *Sav1^f/f^Pgr^Cre/+^* females on day 6 following mLIF treatment on day 5. Scale bars, 100 µm. (E) Representative photographs of uteri from *Sav1^f/f^* females on day 6 of pregnancy, and from *Sav1^f/f^Pgr^Cre/+^* females on day 6 and day 7 of pregnancy, following mLIF treatment on day 4 and day 5, respectively. (F) In situ hybridization of decidual markers *Bmp2* and *Wnt4* in implantation sites from *Sav1^f/f^* females on day 6 of pregnancy, and from *Sav1^f/f^Pgr^Cre/+^* females on day 7 following mLIF treatment on day 5. Scale bars, 500 µm. (G) IF of FLK1 and ZO‐1 in implantation sites from *Sav1^f/f^* (day 6) and *Sav1^f/f^Pgr^Cre/+^* (day 7, following mLIF on day 5) females, showing blood vessels invading the PDZ in *Sav1^f/f^Pgr^Cre/+^* mice as indicated by arrows. White dashed lines indicate primary decidual zones. Scale bars, 200 µm. (C), (D), (F), and (G) Asterisks indicate the location of embryos. M, mesometrial pole; AM, antimesometrial pole.

Leukemia inhibitory factor (LIF), which is transiently expressed in the uterine glands, is indispensable for blastocyst implantation in mice [25, 26, 27]. Notably, intraperitoneal administration of LIF on day 4 of pregnancy restores embryo implantation in mice with defective gland function. To elucidate whether the absence of uterine glands is the sole cause of embryo implantation defect in *Sav1^f/f^Pgr^Cre/+^* mice, we employed a recombinant mouse leukemia inhibitory factor (mLIF) supplementation strategy (Figure 3A). Unexpectedly, administration of mLIF on day 4 failed to rescue implantation, whereas treatment on day 5 successfully promoted blastocyst attachment on the following day (Figure 3B and Table S1). 3D imaging revealed that, following mLIF supplementation on day 5, luminal epithelium of *Sav1^f/f^Pgr^Cre/+^* mice formed implantation chambers, despite the near‐complete absence of adjacent glandular structures (Figure 3C). Consistently, IF analysis of cyclooxygenase‐2 (COX2), a marker of successful implantation, demonstrated that deferred mLIF treatment restored COX2 expression around the implanted embryos (Figure 3D).

To further assess subsequent decidual responses, we monitored implantation sites on days 7 and 9 of pregnancy following mLIF administration on day 5 in *Sav1^f/f^Pgr^Cre/+^* mice. We found that, although the tissue morphology and number of implantation sites two days after mLIF treatment in *Sav1^f/f^Pgr^Cre/+^* mice were comparable to those of floxed controls on day 6 of normal pregnancy (Figure 3E and Table S1), the decidual markers *Bmp2* and *Wnt4* were markedly reduced in the *Sav1^f/f^Pgr^Cre/+^* mice (Figure 3F). The primary decidual zone (PDZ), an avascular region surrounding the implantation chamber that is formed by stromal cells differentiating into epithelial‐like cells to protect the embryo from harmful agents such as immunoglobulins and immune cells [21, 28], was also abnormal. Co‐staining of the endothelial marker fetal liver kinase 1 (FLK1) and the PDZ marker zonula occludens‐1 (ZO‐1) revealed numerous FLK1‐positive cells with primitive vascular structures in the PDZ of *Sav1^f/f^Pgr^Cre/+^* mice (Figure 3G), indicating that the PDZ formed in these females is defective not only morphologically but also functionally. Consistently, 3D imaging demonstrated impaired development of the implantation chamber structure (Figure S2A). These abnormalities were further reflected in apoptosis patterns, as Cleaved‐Caspase 3‐an indicator of apoptosis critical for embryo‐decidua crosstalk [29]‐was appropriately absent in crypt epithelium surrounding the blastocyst in *Sav1^f/f^Pgr^Cre/+^* mice (Figure S2B). The adverse ripple effects became prominent. Although the status and number of implantation sites four days after mLIF administration on day 5 were superior to those observed following day 4 treatment in *Sav1^f/f^Pgr^Cre/+^* mice (Figure S2C and Table S1), the morphology appeared smaller and more rounded, and decidual responses were further compromised compared with normal day 8 implantation sites of *Sav1^f/f^* mice (Figure S2D,E). We further tested whether early mLIF administration on day 3 or combined E_2_/mLIF treatment on day 4 could rescue the implantation defects in *Sav1^f/f^Pgr^Cre/+^* uteri. However, these exogenous supplementation strategies failed to restore implantation, suggesting that the underlying defects may involve a broader network of factors beyond LIF and estrogen signaling (Table S2). These findings indicate that, in addition to gland loss caused by *Sav1* deficiency, an altered “window” also represents a critical factor contributing to implantation defect.

The *Pgr‐Cre* driver mediates gene deletion across all major uterine cell types in the endometrium, including stromal and epithelial cells. To identify the specific cell type in which *Sav1* functions to regulate receptivity, we generated uterine epithelial‐specific *Sav1* knockout mice (*Sav1^f/f^Ltf^Cre^*) using the *Ltf‐Cre* driver, which initiates deletion in adulthood when the uterine glands are already fully developed. Efficient deletion of *Sav1* in the uterine epithelium was validated by qRT‐PCR on isolated epithelial and stromal compartments (Figure S3A). Surprisingly, *Sav1^f/f^Ltf^Cre/+^* mice exhibited litter size comparable to those of littermate controls (Figure S3B), and further analyses of these mice revealed intact gland development, as well as normal embryo implantation and decidualization (Figure S3C–I). Collectively, these results indicate that *Sav1* in uterine stromal cells plays a pivotal role in orchestrating the timely establishment of the implantation window.

### 2.3 *Sav1* Loss Impairs Uterine Receptivity Independent of Canonical Hippo Signaling

**FIGURE 4:**
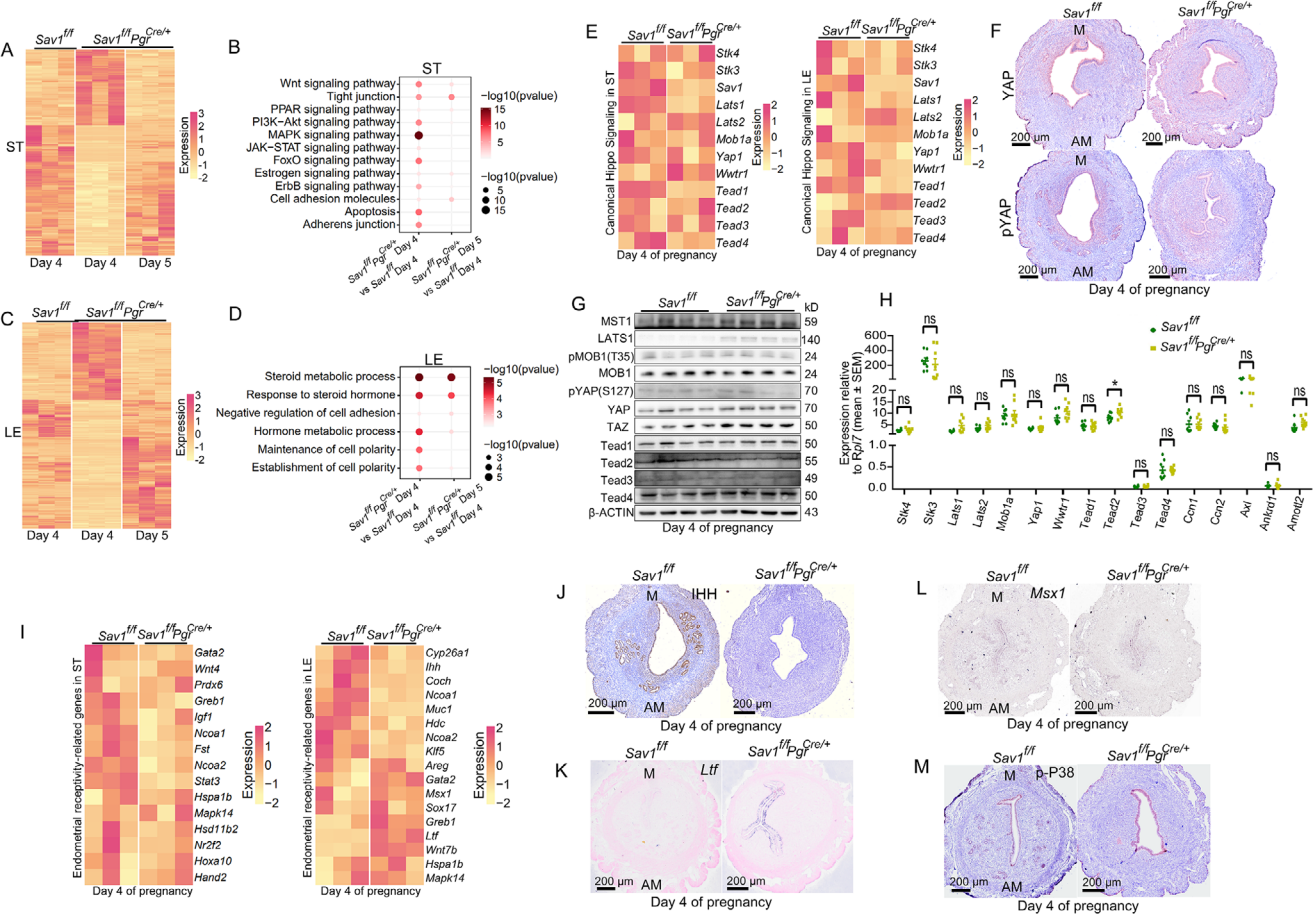
*Sav1* deletion impairs receptivity in a canonical Hippo‐independent manner. (A) Heatmap of gene expression (FPKM) in separated stroma of day 4 pregnant uteri from *Sav1^f/f^*, day 4 and 5 pregnant uteri from *Sav1^f/f^Pgr^Cre/+^* females (*n* = 3). FPKM of each gene from different samples was normalized to Z‐score. FPKM, fragments per kilobase of transcript per million mapped reads. (B) KEGG enrichment analysis of the differentially expressed genes in stroma between *Sav1^f/f^Pgr^Cre/+^* Day 4 and *Sav1^f/f^* Day 4, *Sav1^f/f^Pgr^Cre/+^* Day 5 and *Sav1^f/f^* Day 4. *p* values were generated by clusterProfiler using a hypergeometric test without multiple testing correction. (C) Heatmap of gene expression (FPKM) in separated luminal epithelium of day 4 pregnant uteri from *Sav1^f/f^*, day 4 and 5 pregnant uteri from *Sav1^f/f^Pgr^Cre/+^* females (*n* = 3). (D) KEGG enrichment analysis of the differentially expressed genes in luminal epithelium between *Sav1^f/f^Pgr^Cre/+^* Day 4 and *Sav1^f/f^* Day 4, *Sav1^f/f^Pgr^Cre/+^* Day 5 and *Sav1^f/f^* Day 4. *p* values were generated by clusterProfiler using a hypergeometric test without multiple testing correction. (E) Heatmap showing canonical Hippo pathway gene expression (FPKM) in stroma and luminal epithelium of day 4 pregnant uteri from *Sav1^f/f^* and *Sav1^f/f^Pgr^Cre/+^* females (*n* = 3). (F) IHC analysis of YAP and pYAP on day 4 pregnant uteri from *Sav1^f/f^* and *Sav1^f/f^Pgr^Cre/+^* females. Scale bars, 200 µm. (G) Western blotting of main and active components of Hippo signaling in day 4 *Sav1^f/f^* and *Sav1^f/f^Pgr^Cre/+^* uterine tissues. β‐ACTIN was used as a loading control. (H) qRT‐PCR analysis of upstream core components and key downstream effectors of Hippo signaling in day 4 *Sav1^f/f^* and *Sav1^f/f^Pgr^Cre/+^* uterine tissues (*n* = 8 mice per group). (I) Heatmap showing endometrial receptivity‐related gene expression (FPKM) in stroma and luminal epithelium of day 4 pregnant uteri from *Sav1^f/f^* and *Sav1^f/f^Pgr^Cre/+^* females (*n* = 3). (J–M) IHC or In situ hybridization of IHH (J)*, Ltf* (K), *Msx1* (L), and phospho‐P38 (M) on day 4 pregnant uteri from *Sav1^f/f^* and *Sav1^f/f^Pgr^Cre/+^* females. Scale bars, 200 µm. M, mesometrial pole; AM, antimesometrial pole. Statistical analysis was performed using two‐tailed Student's *t*‐test. Data are presented as mean ± SEM. ns, not significant (*p* > 0.05), ^*^ *p* < 0.05.

Building on these observations, we next sought to elucidate the molecular mechanisms through which *Sav1* regulates uterine receptivity by performing RNA sequencing (RNA‐seq) on isolated epithelial and stromal compartments from uteri of *Sav1^f/f^* mice (day 4) and *Sav1^f/f^Pgr^Cre/+^* mice (days 4 and 5). Notably, the heatmap revealed that the transcriptional profile of *Sav1^f/f^Pgr^Cre/+^* mice on day 4 diverged significantly from that of *Sav1^f/f^*, while on day 5 it more closely resembled the receptive state (day 4 of *Sav1^f/f^*). This finding was further substantiated by Kyoto Encyclopedia of Genes and Genomes (KEGG) enrichment analysis, which revealed markedly altered receptivity‐related pathways in day 4 *Sav1^f/f^Pgr^Cre/+^* uterus compared to day 4 *Sav1^f/f^*. However, these alterations were markedly attenuated in day 5 *Sav1^f/f^Pgr^Cre/+^* uterus, particularly within the endometrial stromal cells (Figure 4A–D). Pairwise comparisons of the transcriptional profiles between three groups of endometrial stromal cells via differential expression analysis, Gene Ontology (GO), and KEGG enrichment analysis indicated substantial dysregulation in receptivity‐related pathways—such as tight junction, cell adhesion molecules, Notch and mitogen‐activated protein kinase (MAPK) signaling, and hormone metabolism—in *Sav1^f/f^Pgr^Cre/+^* mice on day 4 compared to either *Sav1^f/f^Pgr^Cre/+^* on day 5 or *Sav1^f/f^* on day 4. In contrast, few receptivity‐related pathways were enriched when comparing *Sav1^f/f^Pgr^Cre/+^* on day 5 to *Sav1^f/f^* on day 4 (Figure S4).

As SAV1 serves as a critical scaffold protein in the canonical Hippo pathway, its potential involvement in regulating uterine receptivity through Hippo signaling was assessed. RNA‐seq analyses showed no significant alterations in the expression of core Hippo pathway components in epithelial or stromal compartments of *Sav1^f/f^Pgr^Cre/+^* mice on day 4 (Figures 4E and S5A,B). Immunohistochemistry (IHC) further revealed comparable expression of YAP and phosphorylated YAP between genotypes (Figure 4F). Furthermore, western blotting analysis (Figure 4G) and qRT‐PCR (Figures 4H and S5C) likewise showed no notable differences in key Hippo proteins. We wondered whether the gland deficiency caused by SAV1 deficiency is mediated via the Hippo pathway. FOXA2 staining indicated that SAV1 deficiency impairs gland development from postnatal days 7 to 14, despite no significant changes in canonical Hippo pathway components (Figure S6). Together, these data indicate that SAV1 deficiency compromises uterine receptivity as well as gland development through a pathway independent of canonical Hippo signaling.

Given that an appropriate uterine response to ovarian hormones is indispensable for the timely establishment of receptivity, we investigated the expression of hormone‐responsive genes. RNA‐seq analysis on day 4 revealed widespread dysregulation of receptivity‐associated genes in *Sav1^f/f^Pgr^Cre/+^* uteri, characterized by a pronounced downregulation of progesterone‐responsive genes (*Fst*, *Hdc*, *Ihh*, *Cyp26a1*) accompanied by an upregulation of estrogen‐responsive genes (*Ltf*, *Wnt7b*) (Figure 4I). Consistently, the abnormally decreased indian hedgehog (IHH) and increased *Ltf* were observed in *Sav1^f/f^Pgr^Cre/+^* female (Figure 4J,K). Day 3 represents a critical pre‐receptive stage. Driven by a rapid surge in progesterone levels, the uterus enters a transitional state by the evening of day 3 [7]. In contrast to *Sav1^f/f^* mice, *Sav1^f/f^Pgr^Cre/+^* mice displayed abnormal *Ltf* and IHH expression during the pre‐implantation phase, indicative of uterine progesterone resistance. Notably, this expression pattern normalized to a receptive state by day 5 of pregnancy (Figure S7A,B). Furthermore, the expression of the epithelial markers β‐catenin (CTNNB1) and E‐cadherin (CDH1) was indistinguishable between *Sav1^f/f^* and *Sav1^f/f^Pgr^Cre/+^* mice at the pre‐implantation stage, suggesting that uterine epithelial integrity and cell‐cell adhesion are maintained (Figure S7C). These results provide additional evidence that uterine *Sav1* deficiency does not simply cause a generalized delay in implantation, but rather delays the establishment of the receptive uterine state and consequently shifts the functional implantation window toward a later time point. Considering the delayed receptivity in *Sav1^f/f^Pgr^Cre/+^* mice, we further assessed *Msx1* and phosphorylated P38 (p‐P38), both critical for the establishment of uterine receptivity and known to influence implantation timing [3, 30]. However, no significant changes in their expression were detected in the *Sav1^f/f^Pgr^Cre/+^* uteri (Figure 4L,M).

### 2.4 SAV1 Associates With Progesterone Receptor Cochaperone FKBP52 to Coordinate Its Subcellular Localization

**FIGURE 5:**
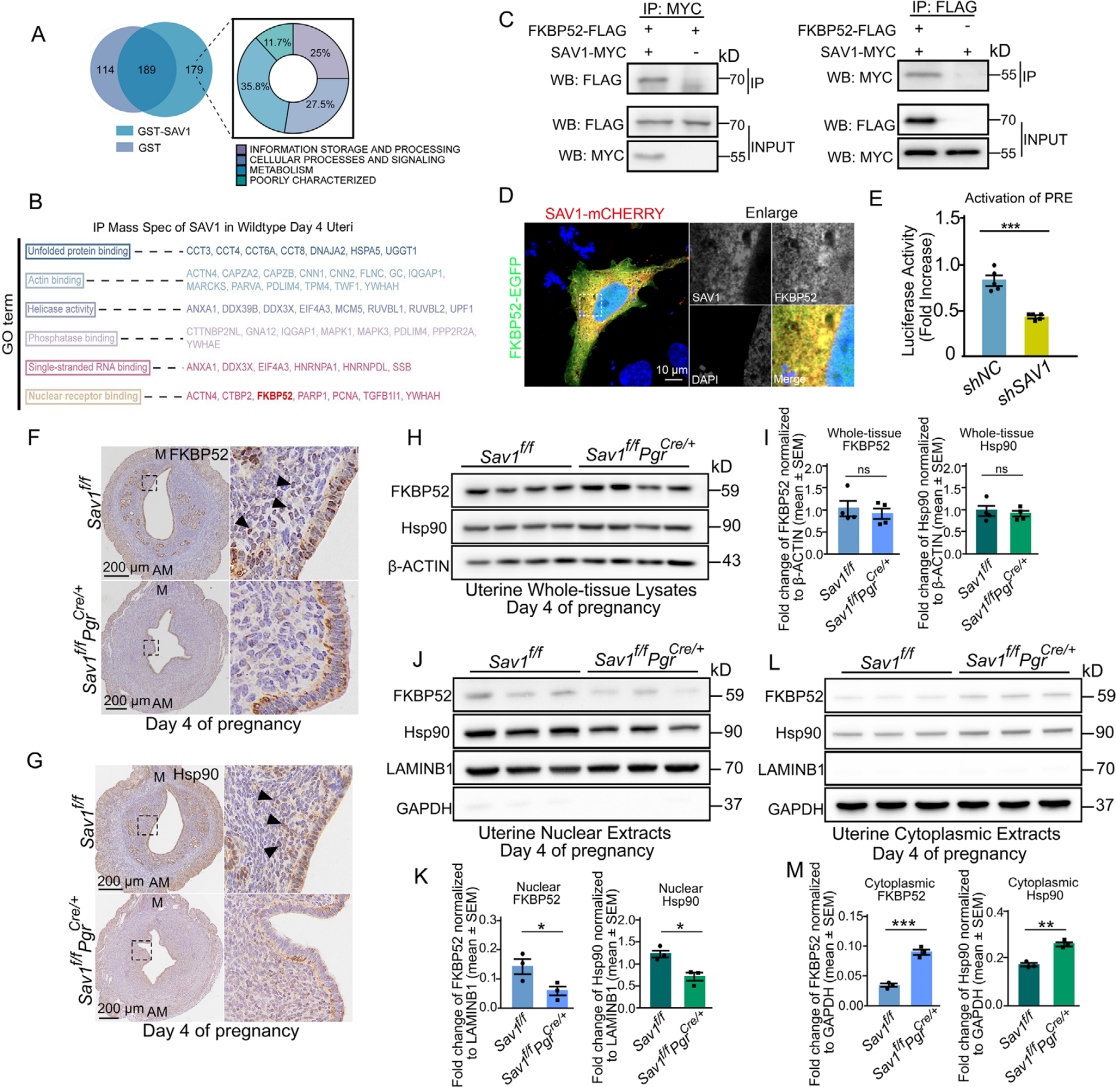
SAV1 interacts with FKBP52 and affects its subcellular localization. (A,B) GST pull‐down/MS identified an interaction between FKBP52 and SAV1 in the day 4 uterus. GST‐pull down was performed on unloaded GST beads and GST beads coupled with SAV1 protein, and uterine proteins of wild‐type female mice on day 4 pregnancy pulled down by them were sent for MS detection respectively. The proteins pulled down by only GST beads coupled with SAV1 were subjected to Clusters of Orthologous Groups annotation (A) and Gene Ontology functional enrichment analysis (B) to identify potential SAV1 interacting proteins that may play a role in early pregnancy. (C) Co‐IP of SAV1 and FKBP52 in 293T cell protein lysates. 293T cells were transiently transfected with SAV1‐MYC and FKBP52‐FLAG and after transfection for 48 h, the whole cell lysates were immunoprecipitated with the anti‐MYC or anti‐FLAG antibody, followed by immunoblotting with the anti‐FLAG or anti‐MYC antibody, respectively. The whole cell lysates were examined by western blotting using the anti‐MYC or anti‐FLAG antibody (input). (D) Colocalization of SAV1‐mCHERRY and FKBP52‐EGFP in isolated hESC cells. Scale bars, 10 µm. (E) Decreased PR‐PRE activation in *SAV1* knockdown hESC cells determined by PRE2‐Tk‐luciferase assay (*n* = 5 for each group). (F,G) IHC of FKBP52 (F) and Hsp90 (G) from *Sav1^f/f^* and *Sav1^f/f^Pgr^Cre/+^* day 4 uteri. Arrowheads indicate nuclear signal in stroma. M, mesometrial pole; AM, antimesometrial pole. Scale bars, 200 µm. (H,I) Western blotting (H) and its quantitative graph (I) of FKBP52 and Hsp90 in *Sav1^f/f^* and *Sav1^f/f^Pgr^Cre/+^* uterine whole tissue lysates (*n* = 4). β‐ACTIN was used as a loading control. (J, K) Western blotting (J) and its quantitative graph (K) of nuclear FKBP52 and Hsp90 in *Sav1^f/f^* and *Sav1^f/f^Pgr^Cre/+^* uterine nuclear extracts (*n* = 3). LAMINB1 and GAPDH were used as nuclear and cytoplasmic loading controls, respectively. (L,M) Western blotting (L) and its quantitative graph (M) of cytoplasmic FKBP52 and Hsp90 in *Sav1^f/f^* and *Sav1^f/f^Pgr^Cre/+^* uterine cytoplasmic extracts (*n* = 3). LAMINB1 and GAPDH were used as nuclear and cytoplasmic loading controls, respectively. Statistical analysis was performed using a two‐tailed Student's *t*‐test. Data are presented as mean ± SEM. ns, not significant (*p* > 0.05), ^*^ *p* < 0.05, ^**^ *p* < 0.01, ^***^ *p* < 0.001.

To define the molecular basis of stromal SAV1‐mediated regulation of progesterone responsiveness, glutathione S‐transferase (GST) pull‐down combined with mass spectrometry (MS) was performed on receptive‐phase uterine tissue to identify potential SAV1‐interacting proteins. A total of 179 proteins specifically bound to SAV1‐GST beads were identified. These proteins were functionally classified by alignment to the Clusters of Orthologous Groups (COG) database. Among the 4 COG groups, the cluster for ‘metabolism’ represented the largest group (35.8%), followed by ‘cellular processes and signaling’ (27.5%) (Figure 5A). GO enrichment analysis of these candidates revealed that SAV1 may regulate endometrial receptivity via interaction with nuclear receptor‐binding proteins. Strikingly, FKBP52, a heat shock protein 90 (Hsp90) cochaperone for steroid hormone receptors that has been implicated in embryo implantation via PR signaling [31, 32], was identified among the top candidates (Figure 5B). We validated the interaction between SAV1 and FKBP52 by co‐immunoprecipitation (Co‐IP) and co‐localization (Figure 5C,D). Given the established role of FKBP52 in modulating steroid hormone receptor transcriptional activity, we next evaluated PR function using a progesterone response element (PRE)‐luciferase reporter assay. Silencing of *SAV1* with *shRNA* in human endometrial stromal cells (hESC) markedly attenuated PR‐driven transcription (Figure 5E).

To elucidate the mechanistic role of SAV1 in regulating FKBP52, we examined the subcellular localization of FKBP52 and its cochaperone Hsp90 via IHC in uteri from *Sav1^f/f^* and *Sav1^f/f^Pgr^Cre/+^* mice on day 4, revealing a marked reduction in nuclear localization of both proteins in epithelial and stromal cells of the *Sav1^f/f^Pgr^Cre/+^* mice (Figure 5F,G). Subsequent western blotting analysis confirmed that total protein levels of FKBP52 and Hsp90 were unchanged, whereas their nuclear expression was specifically reduced in *Sav1^f/f^Pgr^Cre/+^* uteri (Figure 5H–M). Consistently, in *SAV1*‐knockdown hESC, overall FKBP52 and Hsp90 levels were unaltered, yet their nuclear localization was markedly impaired (Figure S8). These results indicate that SAV1 facilitates progesterone responsiveness by promoting the nuclear translocation of PR cochaperones FKBP52 and Hsp90.

### 2.5 SAV1 Promotes FKBP52 Nuclear Translocation by Enhancing Its Phosphorylation by MST Kinase to Regulate PR Activity

**FIGURE 6:**
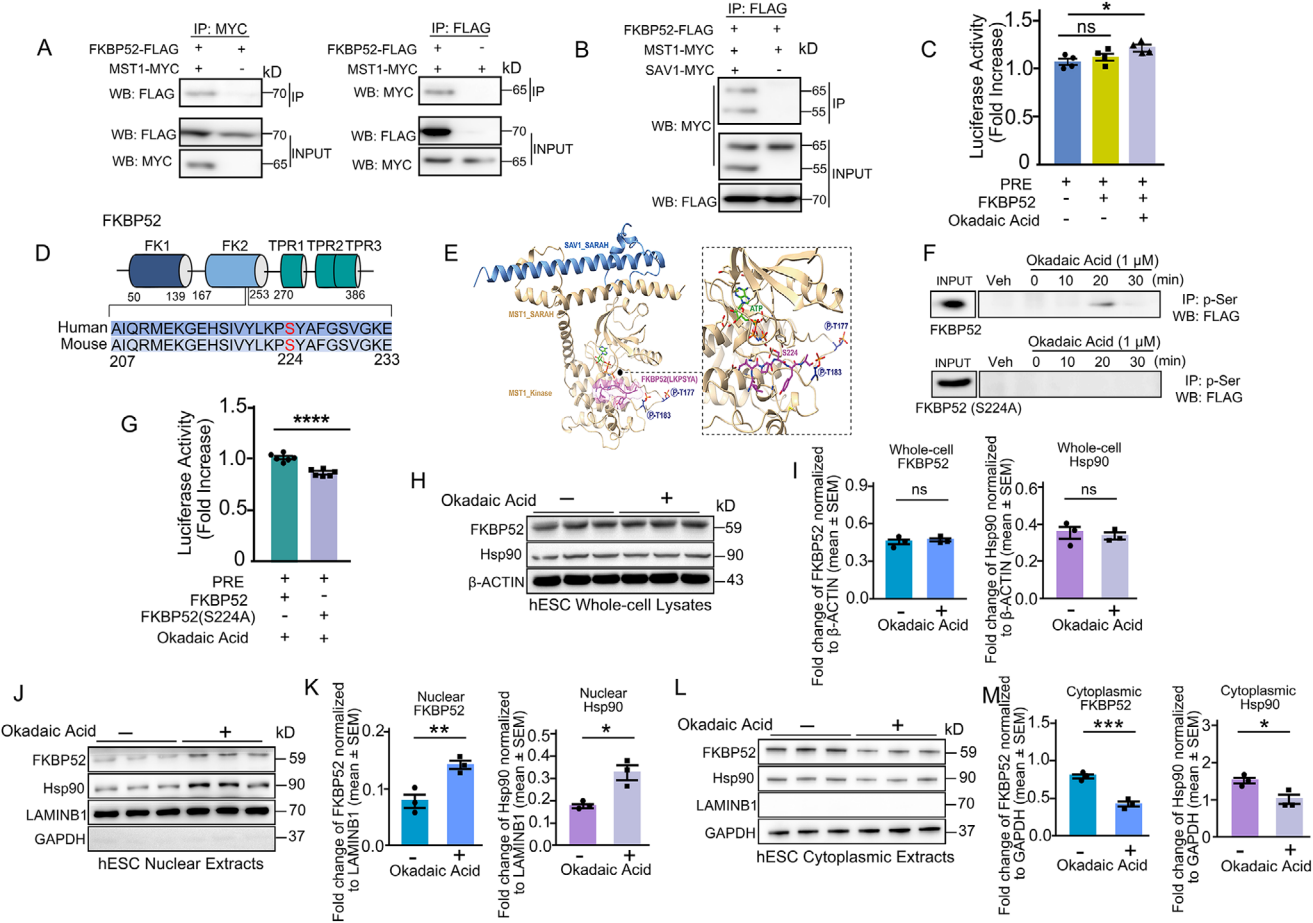
MST1 interacts and phosphorylates FKBP52 to regulate its nucleocytoplasmic shuttling. (A) Immunoblot analysis of MST1‐MYC Co‐IP with FKBP52‐FLAG from lysates of 293T cells transfected with the indicated plasmids. (B) SAV1 promotes the MST1 interaction with FKBP52. Interaction between MST1‐MYC and FKBP52‐FLAG is enhanced with overexpression of SAV1‐MYC in 293T cells. (C) Activated MST1 in conjunction with FKBP52 enhances PR‐PRE activation. Overexpression of FKBP52‐FLAG with 1 µm okadaic acid for 30 min improves PR transcriptional activity in hESC cells. (D) Domain architecture of FKBP52 protein. Serine 224 (red S) resides within the FK2 domain which is conserved between human and mouse. (E) The model of MST1/SAV1 heterodimer in complex with ATP and the substrate FKBP52. MST1 (wheat) and SAV1 (lightblue) form heterodimer depending on SARAH coiled coil domains. The peptide “LKPSYA” of FKBP52 (pink) docks into the active site of MST1 kinase domain. Two phosphorylated residues, T177 and T183 (blue), on the activation loop induce an open conformation to accumulate the substrate. The detailed interaction was illustrated in the dashed box on the right panel. (F) Protein lysates of Ishikawa cells (200 µg) transfected with FKBP52‐FLAG or FKBP52(S224A)‐FLAG are treated with okadaic acid (1 µm) for different periods. The protein lysates are immunoprecipitated with an anti‐p‐Ser antibody, followed by WB with antibodies to FLAG. The results show that only FKBP52‐FLAG can get phosphorylated with MST1 activator OKA and the phosphorylation band is seen at 20 min with a decreased signal at 30 min. (G) PR transcriptional activity in hESC cells with overexpression of FKBP52‐FLAG or FKBP52(S224A)‐FLAG, both treated with 1 µM okadaic acid for 30 min. (H, I) Western blotting (H) and its quantitative graph (I) of FKBP52 and Hsp90 in hESC whole‐cell lysates with or without 1 µm okadaic acid for 30 min (*n* = 3). β‐ACTIN was used as a loading control. (J,K) Western blotting (J) and its quantitative graph (K) of nuclear FKBP52 and Hsp90 in hESC nuclear extracts with or without 1 µm okadaic acid for 30 min (*n* = 3). LAMINB1 and GAPDH were used as nuclear and cytoplasmic loading controls, respectively. (L,M) Western blotting (L) and its quantitative graph (M) of cytoplasmic FKBP52 and Hsp90 in hESC cytoplasmic extracts with or without 1 µM okadaic acid for 30 min (*n* = 3). LAMINB1 and GAPDH were used as nuclear and cytoplasmic loading controls, respectively. In (C), statistical analysis was performed using two‐way ANOVA followed by Dunnett's post hoc test. In (G), (I), (K) and (M), statistical analysis was performed using two‐tailed Student's *t*‐test. Data are presented as mean ± SEM. ns, not significant (*p* > 0.05), ^*^ *p* < 0.05, ^**^ *p* < 0.01, ^***^ *p* < 0.001, ^****^ *p* < 0.0001.

Loss of SAV1 disrupted the nuclear translocation of FKBP52 and Hsp90, implicating potential alterations in FKBP52 post‐translational modifications. SAV1 associates with MST1/2, promoting MST1/2 autophosphorylation and subsequent kinase cascade activation. We employed Co‐IP to investigate the interaction between MST1/2 and FKBP52. The results confirmed that MST1/2 associates with FKBP52, and this interaction is enhanced in the presence of SAV1, suggesting that SAV1 functions as a scaffold to facilitate FKBP52‐MST1/2 complex formation (Figures 6A,B and S9A,B). Consistent with this, treatment with okadaic acid (OKA), a potent activator of MST kinases [33], markedly enhanced PR transcriptional activity in hESC cells overexpressing FKBP52 (Figure 6C).

To further delineate the underlying phosphorylation events, we conducted phosphoproteomic MS following OKA treatment in both *SAV1*‐knockdown and control Ishikawa cells. This analysis identified phosphorylation of FKBP52 at serine 224 (S224) by activated MST1/2, an event abolished upon SAV1 depletion, suggesting that SAV1 facilitates MST‐dependent phosphorylation of FKBP52 at S224 to regulate its subcellular localization (Figure S9C–E).

FKBP52 consists of an N‐terminal FK1 domain with peptidyl prolyl isomerase (PPIase) activity, an enzymatically inactive FK2 domain, and a C‐terminal tetratricopeptide repeat (TPR) domain. The TPR domain mediates binding to Hsp90, whereas the FK1 domain serves both as the enzymatic core and as a docking site that potentiates steroid hormone receptor signaling, with the FK2 domain further contributing to the enhancement of FK1 function [34, 35, 36]. Notably, S224 is located within the FK2 domain and is evolutionarily conserved between human and mouse (Figure 6D). The phosphorylation‐site motif analysis confirmed that the amino acids surrounding S224 exhibit the preferred substrate specificity for the MST1/2 kinase, which belongs to the mitogen‐activated protein kinase kinase kinase (MAP3K) group of the human serine/threonine (Ser/Thr) kinome [37]. To better understand the molecular mechanism by which MST1 phosphorylates FKBP52, we constructed a 3D model of the MST1‐SAV1 heterodimer complexed with the “LKPSYA” peptide of FKBP52 using AlphaFold software. The referenced templates were derived from crystal structures of phosphorylated human MST1 (Protein Data Bank [PDB] ID 3COM), human MST2 in complex with the SAV1 Salvador/RASSF/Hippo (SARAH) domain (PDB ID 6AO5), Ser/Thr kinase PAK4 in complex with the Paktide T peptide substrate (PDB ID 4JDJ), and mouse cyclic adenosine monophosphate (cAMP)‐dependent protein kinase with adenosine triphosphate (ATP) (PDB ID 1ATP). The docking results clearly showed that the “LKPSYA” peptide of FKBP52 was accommodated in the catalytic center of activated MST1, pre‐loaded with ATP (Figure 6E). A similar result was obtained from the predicted model of the MST2‐SAV1 heterodimer in complex with the FKBP52 peptide substrate (Figure S9F). Based on these models and previous biochemical data [14, 38], we propose a potential mechanism. Both SAV1 and MST1 contain a SARAH domain. These coiled‐coil domains readily form heterodimers. In addition, SAV1 possesses tandem WW domains, which facilitate domain‐swapped SAV1 dimer formation. As a result, two MST1/2‐SAV1 heterodimers associate to form a tetramer. The close proximity of the two MST1/2 molecules promotes auto‐activation, leading to phosphorylation of a key residue‐T183 in MST1 or T180 in MST2. This phosphorylation induces an open conformation in the activation loop, thereby fully activating the kinase domain of MST1/2. Finally, the activated MST1/2 recognizes the preferred motif of FKBP52 and phosphorylates S224.

These results prompted us to investigate whether Ser224 of FKBP52 serves as a phosphorylation site downstream of MST1/2 activation. IP‐kinase assays in Ishikawa cells expressing either wild‐type or S224‐mutant FKBP52 revealed that the S224 mutant exhibited markedly reduced serine phosphorylation following OKA‐induced MST1/2 activation (Figure 6F). Consistently, luciferase reporter assays showed that the S224 mutation abrogated MST‐mediated phosphorylation and significantly diminished PR‐dependent transcription compared to wild‐type FKBP52 (Figure 6G). Moreover, OKA‐induced MST activation enhanced nuclear accumulation of FKBP52 and Hsp90 without substantially altering their total protein levels (Figure 6H–M). Collectively, these findings indicate that MST‐mediated phosphorylation of FKBP52 at S224 promotes its nuclear localization and enables its function as a PR cochaperone, thereby supporting PR‐dependent transcriptional regulation. SAV1 is critical in this process, acting as an adaptor that facilitates the recruitment of FKBP52 to MST1/2.

### 2.6 Uterine Quiescence Normalizes Window Shift and Improves Implantation in Reactivated *Sav1^f/f^Pgr^Cre/+^* Mice

Implantation failure in *Fkbp52* null mice was observed as a consequence of uterine progesterone resistance; however, progesterone supplementation could rescue implantation and decidualization in a manner dependent on genetic background [32]. We administered exogenous progesterone to *Sav1^f/f^Pgr^Cre/+^* mice from day 1 to day 4 of pregnancy and additional mLIF on day 4; however, this treatment failed to rescue embryo implantation on day 5, although a partial improvement in implantation was observed compared with untreated *Sav1^f/f^Pgr^Cre/+^* mice (Table S3). This effect may be attributable to the C57BL6/129 genetic background of *Sav1^f/f^Pgr^Cre/+^* mice, which renders them relatively insensitive to progesterone.

**FIGURE 7:**
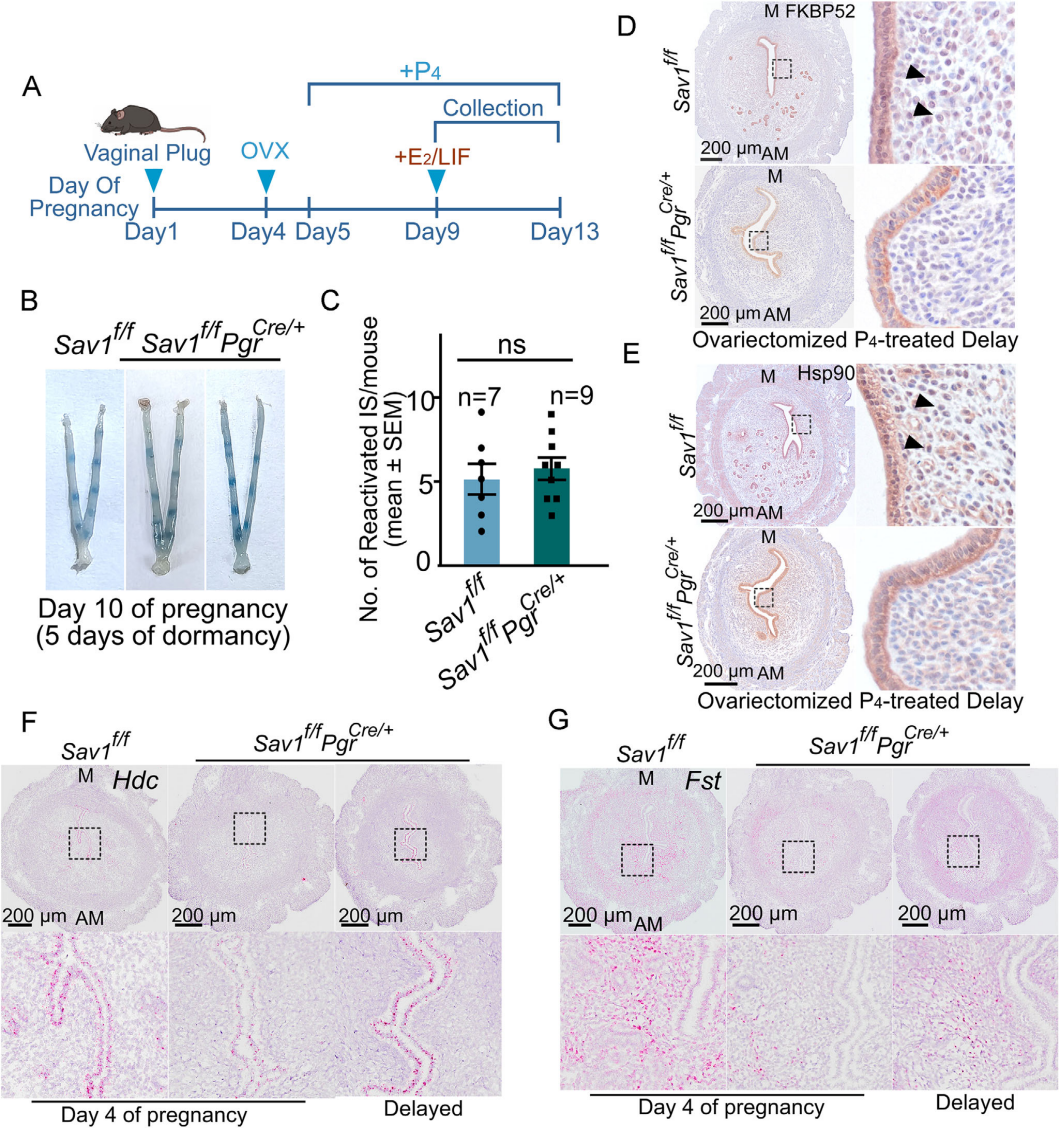
Uterine quiescence restores “window” of implantation in *Sav1^f/f^Pgr^Cre/+^* mice. (A) The scheme shows the schedule of inducing the delayed implantation model and evaluating the implantation events. On the morning of day 4 of pregnancy, plug‐positive females were ovariectomized and then administered P_4_ daily (2 mg, subcutaneously) from day 5 until the day prior to tissue collection. E_2_ (25 ng, subcutaneously) and mLIF (50 µg, intraperitoneally) were injected on day 9 to induce embryo implantation after 5 days of dormancy. (B) Reactivated implantation is comparative between *Sav1^f/f^* and *Sav1^f/f^Pgr^Cre/+^* females under delayed implantation circumstances. (C) The number of reactivated implantation sites are assessed in *Sav1^f/f^* (*n* = 7) and *Sav1^f/f^Pgr^Cre/+^* (*n* = 9) females. (D,E) IHC of FKBP52 (D) and Hsp90 (E) in ovariectomized P_4_‐treated delayed uteri from *Sav1^f/f^* and *Sav1^f/f^Pgr^Cre/+^* females. Scale bars, 200 µm. Arrowheads indicate nuclear signal in stroma. (F,G) RNAscope shows lower transcriptional levels of P_4_ responsive genes *Hdc* (F) and *Fst* (G) in *Sav1^f/f^Pgr^Cre/+^* day 4 uteri compared to *Sav1^f/f^*, and delayed uteri in *Sav1^f/f^Pgr^Cre/+^* mice partially restored their expression. Scale bars, 200 µm. M, mesometrial pole; AM, antimesometrial pole. Statistical analysis was performed using two‐tailed Student's *t*‐test. Data are presented as mean ± SEM. ns, not significant (*p* > 0.05).

Delayed implantation, also known as embryonic diapause, is a physiological phenomenon in certain mammals wherein blastocysts enter a state of dormancy upon entering the uterine cavity rather than implanting immediately, later resuming development and implanting after a brief period‐a process beneficial for reproductive success [39, 40]. In mice, bilateral ovariectomy on day 4 of pregnancy eliminates endogenous estrogen and progesterone, and exogenous progesterone administration maintains blastocysts in a dormant state without initiating implantation until an estrogen surge triggers activation. We attempted an embryonic diapause‐reactivation strategy to rescue embryo implantation defect in *Sav1^f/f^Pgr^Cre/+^* mice, aiming to provide an optimal period for progesterone receptor responsiveness to P_4_ in the *Sav1^f/f^Pgr^Cre/+^* uteri. We induced uterine quiescence for five days in both *Sav1^f/f^* and *Sav1^f/f^Pgr^Cre/+^* mice, followed by estrogen and mLIF administration, and subsequently assessed embryo attachment and decidualization at predetermined time points (Figure 7A). Interestingly, uterine quiescence did not correct the *Sav1* deficiency or restore SAV1 expression (Figure S10A,B), the number and quality of implantation sites in *Sav1^f/f^Pgr^Cre/+^* mice on day 10 were comparable to those in *Sav1^f/f^* mice uteri (Figure 7B,C and Table S4), indicating that *Sav1^f/f^Pgr^Cre/+^* mice maintain an implantation window temporally synchronized with *Sav1^f/f^* mice.

We next examined the subcellular localization of FKBP52 and Hsp90 by IHC. After 5 days of uterine quiescence, their nuclear localization showed no marked change compared with the previous state, remaining lower in *Sav1^f/f^Pgr^Cre/+^* uteri than in *Sav1^f/f^* (Figure 7D,E). RNAscope analysis confirmed that expression of the P_4_‐responsive genes *Hdc* and *Fst*—which were reduced in *Sav1^f/f^Pgr^Cre/+^* mice on day 4—was restored following 5 days of uterine quiescence (Figure 7F,G). Despite the persistence of structural gland defects, delayed implantation substantially restored the progesterone resistance program, as evidenced by the normalized expression of key glandular and hormone‐responsive genes (Figure S10C,D). These findings indicate that the extended duration of progesterone treatment during diapause allows sufficient time to overcome P_4_‐PR signaling defects in *Sav1^f/f^Pgr^Cre/+^* mice. We further evaluated decidualization following reactivation, implantation site numbers, and 3D architecture as well as expression of decidual markers on day 11 (5 days of dormancy) were comparable between *Sav1^f/f^* and *Sav1^f/f^Pgr^Cre/+^* mice under identical treatment conditions (Figure S10E–G and Table S4). Notably, the restorative effect of delayed implantation on decidualization in *Sav1^f/f^Pgr^Cre/+^* mice persisted even four days post‐reactivation under continuous progesterone support, as reflected by similar implantation site number and morphology, as well as unaltered expression of the decidual markers *Prl8a2* and *Prl3c1* (Figure S10H–J and Table S4). These results indicate that *Sav1* deficiency diminishes P_4_‐PR responsiveness, delaying the P_4_‐primed uterus from entering a pre‐receptive (transitional) state, which initiates uterine receptivity in the presence of coordinated estrogen signaling upon this transitional phase. As E_2_‐mediated embryo reactivation and implantation occur through LIF induction, LIF administration alone is expected to recapitulate this process (Table S5). Although exogenous LIF supplementation effectively restored embryo implantation, pregnancy could not be sustained to term in the absence of glandular structures, indicating that uterine glands are indispensable for maintaining mid‐ to late‐stage gestation [25]. Nevertheless, despite synchronization of the implantation window between *Sav1^f/f^Pgr^Cre/+^* and *Sav1^f/f^* mice through delayed implantation and reactivation, the lack of glandular structures in *Sav1^f/f^Pgr^Cre/+^* uteri precluded further evaluation of their reproductive outcomes.

To investigate the involvement of *SAV1* in the pathophysiology of human pregnancy complications, we reanalyzed publicly available single‑cell transcriptomic data of the human endometrium, including samples from patients with recurrent implantation failure (RIF) and fertile controls (GSE250130) [41]. This reanalysis revealed that *SAV1* expression in endometrial stromal cells was significantly lower in RIF patients compared to fertile women. Moreover, the expression of progesterone‑responsive genes was also markedly reduced in the endometrium of RIF patients. To further extend these observations, we analyzed additional publicly available single‑cell sequencing datasets from patients with recurrent miscarriage (PRJNA672658 and GSE214607) [42, 43]. Consistent with the findings in RIF, both *SAV1* and progesterone‑response gene signatures were significantly downregulated in the decidual tissues of recurrent miscarriage patients (Figure S11). Collectively, these analyses of public human datasets provide preliminary clinical evidence that aligns closely with our mouse model results. The consistency across human samples and animal models strongly supports a critical role for SAV1 in pregnancy and reinforces the clinical relevance of the SAV1–PR signaling axis proposed in this study.

## 3 Discussion

On‐time embryo implantation is critical for successful pregnancy, with uterine receptivity being an essential prerequisite. Although the establishment of uterine receptivity is predominantly regulated by ovarian‐derived steroid hormones, the molecular mechanisms by which the P_4_‐PR signaling pathway precisely modulates stage‐specific functions in the P_4_‐primed uterus, particularly to facilitate effective uterine preparation for reaching the transitional phase, remain to be elucidated. This study reveals that SAV1, a core member of Hippo signaling, serves as a key determinant of uterine PR actions in preparing the uterus receptivity.

Previous studies have demonstrated that the Hippo signaling pathway is involved in decidualization, as depletion of *Yap1* and *Wwtr1* by *Pgr‐Cre* driver abolishes the decidual response [19]. In contrast, we found that *Sav1^f/f^Pgr^Cre/+^* females display near‐complete infertility, characterized by the loss of uterine glands and implantation defect. This phenotypic discrepancy suggests that SAV1, an upstream scaffold protein of the Hippo kinase cascade, may regulate uterine development and function not solely through YAP/TAZ signaling but also via additional mechanisms. More importantly, our findings reveal that *Sav1^f/f^Pgr^Cre/+^* mice fail to achieve embryo implantation following exogenous mLIF administration on day 4, whereas implantation is observed within 24 h after mLIF injection on day 5. This indicates that abnormal glandular function alone does not fully account for the implantation defects caused by *Sav1* deficiency, which are further characterized by a significant temporal delay in the establishment of uterine receptivity in *Sav1^f/f^Pgr^Cre/+^* mice. Although implantation sites with a normal appearance were observed in *Sav1^f/f^Pgr^Cre/+^* uteri when mLIF was given on day 5 of pregnancy, implantation chambers showed no further development and exhibited impaired decidualization thereafter, indicating that the altered implantation window is insufficient to support effective bidirectional communication between the embryo and *Sav1^f/f^Pgr^Cre/+^* uteri. Furthermore, female mice with uterine epithelial *Sav1* deletion exhibit apparently normal pregnancy outcomes, our findings indicate that the *Sav1* in the uterine stromal cells may determine the onset of uterine preparation to receptivity.

The Hippo signaling pathway associates with steroid hormone receptors—including estrogen receptor (ER) and androgen receptor (AR)—to participate in hormone‐dependent tumorigenesis, particularly in breast and prostate cancers [44, 45, 46]. Given this established interaction between Hippo components and steroid receptors, we asked whether Hippo signaling might similarly influence PR activity in the receptive endometrium. Intriguingly, our study reveals that *Sav1*‐deficient endometrial tissue on day 4 exhibits reduced PR activation without concomitant alterations in canonical Hippo downstream signaling at either the transcriptional or protein level. These findings suggest that the temporal dysregulation of “window” establishment caused by *Sav1* deficiency occurs independently of canonical Hippo signaling, while concurrently eliciting P_4_ resistance.

Moreover, we discovered that SAV1 physically interacts with FKBP52, a cochaperone of Hsp90, is well established as a critical determinant of PR signaling, with null mice exhibiting implantation defects. FKBP52 is a positive regulator of the glucocorticoid receptor (GR), PR, AR, but not ER [31, 47, 48]. Protein–protein interaction analysis has demonstrated that steroid receptors associate with the Hsp90‐FKBP52 complex in the cytoplasm. Within this complex, Hsp90 maintains the receptors in a properly folded and “activation‐competent” state, while FKBP52, as an immunophilin, interacts with both the cytoskeleton and motor proteins [34, 35]. The N‐terminus of FKBP52 directly engages dynein and components of the dynactin complex, thereby loading the receptor‐Hsp90‐FKBP52 complex onto microtubule tracks and enabling its retrograde transport from the cytoplasm toward the nucleus under the driving force of dynein [36, 49]. In vitro analysis suggests Hsp90‐FKBP52 complex facilitates the rapid, steroid‐dependent nuclear accumulation of cytoplasmic steroid receptors. Although FKBP52 has long been recognized as a key factor involved in the nuclear import of steroid hormone receptors, no significant alterations in PR nuclear localization were observed in the uteri of *Fkbp52*‐deficient mice [32, 50], and similarly, our study did not detect any overt changes in PR nuclear localization in *Sav1*‐deficient uteri, indicating that the mechanisms underlying Hsp90‐FKBP52‐mediated regulation of P_4_‐PR signaling require further investigation.

In parallel, SAV1 facilitates MST1/2 activation by promoting their dimerization and autophosphorylation and maintaining their active conformation. Our mechanistic analyses reveal that MST1/2‐SAV1‐mediated phosphorylation of FKBP52 at S224 is critical for its nuclear import along with Hsp90, facilitating PR‐driven transcription. Interestingly, S224 is located within the FK2 domain, a region of previously unclear function [35, 51], and our results indicate that FK2 contributes to the cytoplasm‐to‐nucleus translocation of FKBP52. Currently, direct in vivo evidence for a causal role of FKBP52 S224 phosphorylation remains lacking. Future studies using specific phospho‐antibodies will be required to further investigate and validate this regulatory axis in vivo. Nevertheless, these findings place SAV1 at the center of a noncanonical signaling pathway, in which it functions not through its classical role in canonical Hippo signaling but rather as a scaffold that bridges MST kinases with steroid receptor cochaperones. This mechanism expands the repertoire of regulatory inputs governing hormone‐dependent biological processes, demonstrating that Hippo signaling, via the MST1/2–SAV1 complex, modulates the steroid receptor cochaperone FKBP52 through a noncanonical route to exert its function.

Our study indicates that SAV1 deficiency does not globally inactivate canonical Hippo signaling, as core pathway components and YAP phosphorylation remained largely unchanged in *Sav1*‐deficient uteri. Rather, SAV1 likely acts as a context‐dependent scaffold facilitating MST1/2‐substrate interactions independent of its canonical role. This notion is consistent with previous reports showing that canonical Hippo signaling is dispensable for uterine receptivity, whereas *Yap1/Wwtr1* deletion primarily affects decidualization [19, 20]. During the peri‐implantation period, the endometrium undergoes highly dynamic physiological changes. Prior to implantation, stromal cells establish receptivity in response to estrogen and progesterone; post‐implantation, they differentiate into decidual cells to support embryo development. Collectively, our findings suggest that the SAV1‐dependent MST1/2–FKBP52 module primarily modulates pre‐implantation stromal cell responsiveness to P_4_, while canonical Hippo signaling predominantly drives decidualization. Notably, although delayed implantation partially restored decidual capacity in our model, the artificial decidualization assay revealed that SAV1 deficiency still significantly impacts this process.

Importantly, induction of delayed implantation and reactivation with exogenous LIF normalized receptivity in *Sav1^f/f^Pgr^Cre/+^* uteri, restoring implantation rates and decidualization markers to levels comparable with controls. Our findings suggest that uterine gland defects and progesterone resistance contribute to implantation defect at distinct but interconnected stages. Impaired gland development compromises the secretion of gland‐derived factors, particularly LIF, thereby impairing initial blastocyst attachment. In contrast, attenuated progesterone responsiveness limits the establishment of endometrial receptivity. Notably, despite the persistence of structural gland defects, delayed implantation significantly rescues the receptivity abnormalities caused by *Sav1* deficiency. This phenomenon indicates that extending uterine quiescence can re‐establish the temporal coordination of progesterone signaling. Consequently, it overcomes certain functional receptivity defects without correcting the underlying glandular morphological abnormalities. These findings support a model in which gland defects and progesterone resistance cooperate during implantation but predominantly affect different phases of the process. This rescue demonstrates that uterine quiescence can mitigate temporal misalignment of hormone responsiveness and underscores the central role of stromal *Sav1* in synchronizing uterine readiness with embryonic development (Figure S12).

By identifying MST1/2‐SAV1‐dependent phosphorylation as a regulatory mechanism for FKBP52 function, we provide a new molecular entry point for understanding the timely establishment of the implantation window. Our findings demonstrate that modulation of uterine quiescence can restore temporally misaligned endometrial receptivity in a preclinical model, highlighting the intrinsic plasticity of the endometrium and offering proof‐of‐concept for therapeutic intervention. In clinical settings such as assisted reproductive technology, controlled ovarian hyperstimulation often perturbs endometrial timing. Based on our results, prolonging progesterone exposure to reset the implantation window may represent a potential strategy to improve implantation outcomes in patients undergoing hormone replacement cycles who experience implantation defect or unexplained early miscarriage. Mechanistically, by identifying a Hippo pathway scaffold protein as a regulator of nuclear receptor co‐regulator function, this study expands the conceptual framework underlying the dynamic control of uterine receptivity.

Notably, the precise mechanism underlying the glandular deficiency in *Sav1^f/f^Pgr^Cre/+^* mice remains unclear. Our study found that during gland development, the canonical Hippo pathway exhibited no significant differences between *Sav1^f/f^Pgr^Cre/+^* and *Sav1^f/f^* mice. This result is consistent with the normal gland development phenotype observed in *Pgr‐Cre*‐mediated *Yap1* and *Wwtr1* double‐knockout mice [19]. Furthermore, FKBP52‐ and PR‐deficient mice also retain intact glandular architecture [10, 31, 52]. Collectively, these lines of evidence suggest that SAV1 regulates uterine glandogenesis independently of these canonical pathways, and the underlying mechanism warrants further investigation in future studies.

## 4 Experimental Section

### 4.1 Mice

*Pgr^Cre/+^* (#017915) [53] and *Ltf^Cre/+^* (#026030) [54] mouse lines were obtained from Jackson Laboratories. *Sav1^f/f^* (T008300) mice were purchased from GemPharmatech (Nanjing, China). *Sav1^f/f^Pgr^Cre/+^* and *Sav1^f/f^Ltf^Cre/+^* mice were generated by mating *Sav1^f/f^* females with *Pgr^Cre/+^* and *Ltf^Cre/+^* males, respectively. All mice used in this study were housed in barrier facilities at Shandong University's Animal Care Facility according to the National Institutes of Health Guide for the Care and Use of Laboratory Animals, with the approval of the Institutional Animal Care and Use Committee of Shandong University (Permit No. SDU‐IACUC‐25066).

### 4.2 Analysis of Pregnancy Events

Adult female mice of two conditional knockout models were housed with fertile wild‐type male mice overnight to induce pregnancy. The morning of finding the presence of a vaginal plug was considered successful mating (day 1 of pregnancy). Litter size and pregnancy rate were monitored for the whole pregnancy process. To confirm that plug‐positive mice were pregnant on day 4 of pregnancy, one uterine horn was flushed with saline to detect blastocyst existence. For day 5 and day 6 pregnant mice, 100 µL of 1% Chicago Blue in saline was injected intravenously to visualize implantation sites as blue bands. If no blue band was observed, uterine horns were flushed to check the presence of embryos. For recombinant mouse LIF treatment, each mouse was given an intraperitoneal injection of 50 µg at 9:00 a.m. on day 3 evening, day 4 morning, or day 5 morning of pregnancy. For preimplantation supplementation with P_4_ or E_2_, E_2_ was administered subcutaneously at 25 ng on day 4 together with mLIF; P_4_ was administered subcutaneously at a dose of 2 mg each morning from day 1 to day 4, with mLIF co‐administered on the morning of day 4. All mice were sacrificed on day 5 for implantation evaluation. To induce delayed implantation, pregnant mice on day 4 morning were ovariectomized bilaterally and injected with progesterone (P_4_, 2 mg/mouse) subcutaneously from day 5 until the day of evaluation. For activation of blastocyst with termination of delay, estradiol‐17β (E_2_, 25 ng/mouse) and mLIF (50 µg/mouse) were injected on day 9 subcutaneously and intraperitoneally respectively. Mice were killed on day 9 before activation or on day 10, 11, and 13 after E_2_ injection (equivalent to day 5, 6, and 8 of normal implantation). All steroids were dissolved in sesame oil.

### 4.3 Artificial Decidualization Response in Mice

Bilateral ovariectomy was performed, and mice were left untreated for 14 days to allow clearance of endogenous hormones. Mice then received daily subcutaneous injections of E_2_ (100 ng/mouse) for 3 days, followed by 2 days without treatment. Subsequently, mice were injected daily with P_4_ and E_2_ (100 µL of a solution containing 10 mg/mL P_4_ and 67 ng/mL E_2_ in sesame oil). On the afternoon of the third combined P_4_/E_2_ injection, approximately 6 h after the injection, 50 µL of sesame oil was injected into the lumen of one uterine horn as a decidual stimulus to mimic embryo apposition. Daily P_4_/E_2_ injections were continued until uterine tissues were collected 5 days after the decidual stimulus for histological and RNA analyses.

### 4.4 Recombinant Mouse LIF

The gene coding leukemia inhibitory factor (LIF) was cloned into pGEX‐6p‐1 vector with a GST tag at the N terminus. The plasmid was transformed into *E. coli* BL21(DE3) cells. Protein expression and purification were performed as previously described 50 with minor modifications. Briefly, a single colony was inoculated into Luria‐Bertani (LB) medium containing 100 µg/mL ampicillin and grown at 37°C until the OD600 reached 0.6–0.8. Protein expression was induced by adding 0.5 mM isopropyl β‐d‐1‐thiogalactopyranoside (IPTG), and the culture was further incubated at 18°C for 16 h. The cells were harvested by centrifugation and lysed by sonication in lysis buffer (20 mm Tris‐HCl pH7.5, 150 mM NaCl and 2% Glycerol). The clarified supernatant was loaded onto a Glutathione Sepharose 4B column, and the purified protein was eluted with lysis buffer containing additional 50 mm reduced glutathione. The purified protein was dialyzed and stored at −80°C.

### 4.5 Immunofluorescence

For uterine tissues, they were mounted on the specimen table using semi‐frozen optimal cutting temperature (OCT compound (Sakura, 4583) and then sliced at −20°C using a low‐temperature freezing microtome (Leica, CM1860). Sections with 12 µm thickness were mounted onto a poly‐L‐lysine coated slide. Tissue sections from control and experimental groups were processed onto the same slide and then fixed in cold 4% paraformaldehyde (PFA) (Sigma, 158127–500) for 10 min after air drying. Afterward, the sections were washed with 1×PBST (PBS+0.1% Triton X‐100). For hESC cells, they were plated on glass coverslips in 12‐well plates. Similarly, they were fixed with 4% PFA for 10 min at room temperature and washed with PBS for three times. Tissues and cells were both blocked using a 3% bovine serum albumin (BSA) solution and incubated with the indicated primary antibodies at 4°C overnight. Primary antibodies and corresponding secondary antibodies conjugated with Alexa Fluor 488 or 594 were then applied for visualization. Nuclear staining was carried out using DAPI (Solarbio, C0065). Images were captured using a panoramic scanning fluorescence microscope (OLYMPUS, VS120) and processed using OlyVIA viewing software for tissue sections. As for cell IF images, a Zeiss Airyscan confocal microscope (Zeiss, LSM900) was used to capture and ZEN viewing software was used to process. The antibodies utilized in this study are listed in *SI Appendix*, Table S6.

### 4.6 Immunohistochemistry

Paraffin tissues were fixed in 10% neutral formalin, subjected to gradient ethanol dehydration treatment. Antigen retrieval was carried out using citrate sodium buffer (pH 6.0) or Tris‐EDTA buffer (pH 9.0) for 20 min in boiling water. Subsequently, tissues were treated with an endogenous peroxidase blocking solution (Beyotime, P0100A) and were then blocked with 3% BSA for 60 min at room temperature, followed by incubation with primary antibodies. The chromogenic reaction was visualized using a DAB kit (Zhongshan Golden Bridge Bio‐technology, ZLI‐9018). Images were captured and analyzed using a panoramic scanner bright field microscope (OLYMPUS, VS120) and OlyVIA viewing software.

### 4.7 RNA Isolation and qPCR

RNA from tissues in TRIzol (Invitrogen, 15596018) was extracted by chloroform and isopropanol. The RNA concentration and purity were measured using a UV–vis Spectrophotometer (Thermo, NanoDrop OneC). Up to 1 µg total RNA after removing genomic DNA from each sample was reverse transcribed with an Evo M‐MLV RT Kit (Accurate Biology, AG11711). qRT‐PCR was performed with SYBR Green Master Mix (Vazyme, Q311‐02) and analyzed using a Real‐Time PCR system (Light Cycler 96) with specific primers listed in *SI Appendix*, Table S7. *Rpl7* served as an internal control and the relative mRNA level of each gene was calculated using the ΔCt method.

### 4.8 Luminal Epithelial and Stromal Cells Separation

The uterine tissues from mice of different genotypes were extracted and cut into 2–3 mm fragments. Pancreatin (Sigma, P3292) and Dispase (Roche, 4942078001) were used to digest uterine fragments at 37°C for 30 min. Subsequently, the epithelial layer was carefully separated from the inside part of the tissue fragments under a stereomicroscope, followed by rinsing with 1×PBS. The remaining tissue fragments were subjected to Accumax (Sigma, A7089) digestion at 37°C for 30 min and vigorously shaked every 5 min. The mixture was filtered through a nylon mesh to remove excess cell mass and then centrifuged to collect isolated uterine stromal cells.

### 4.9 RNA Extraction Library Construction and Sequencing

Luminal epithelial and stromal cells were separated from *Sav1^f/f^* day 4 uteri, *Sav1^f/f^Pgr^Cre/+^* day 4 and day 5 uteri, respectively. Isolated epithelial and stromal tissues were stored separately at −80°C and subsequently shipped under cold chain conditions to LC‐BIO Technologies (Hangzhou) Corporation for RNA extraction and sequencing. Total RNA was extracted with TRIzol (Invitrogen, 15596018), and integrity was verified (RIN > 7.0) using an Agilent Bioanalyzer 2100. Poly(A) mRNA was enriched from 5 µg RNA using Dynabeads Oligo(dT), fragmented (94°C, 5–7 min; NEB #e6150), and reverse‐transcribed with SuperScript II. Second‐strand synthesis was performed using *E. coli* DNA polymerase I, RNase H, and dUTP. cDNA was A‐tailed, adapter‐ligated, size‐selected, and PCR‐amplified (8 cycles) following UDG treatment. Final libraries (insert size 300 ± 50 bp) were sequenced on an Illumina Novaseq 6000 (PE150).

### 4.10 Bioinformatics Analysis

The single‐cell RNA sequencing (scRNA‐seq) data of the mouse uterus on day 4 of pregnancy were downloaded from Gene Expression Omnibus database under accession number GSE275806. The standard workflow of Seurat (version 5.0.1) was utilized in scRNA‐seq data analysis. Cells with fewer than 500 detected genes. Genes expressed in fewer than three cells were removed.

The scRNA‐seq datasets of endometrium from patients with RIF were downloaded from Gene Expression Omnibus database under accession number GSE250130; scRNA‐seq datasets of the decidua from patients with recurrent miscarriage (RM) were downloaded from Gene Expression Omnibus database under accession number GSE214607 and the National Genomics Data Center under accession number PRJNA672658. The standard workflow of cell clustering in Seurat was utilized. Cells with fewer than 500 detected genes. Genes expressed in fewer than three cells were removed. To identify differentially expressed genes among groups, the FindMarkers function with the Wilcoxon rank‐sum test algorithm was used under the following criteria: logfc. threshold > 0.25, min.pct > 0.25.

RNA‐seq raw data were filtered to obtain clean data after by using Cutadapt (version 1.9). High‐quality clean data were aligned to the mouse reference genome GRCm39 using HISAT2 (version 2.2.1). Differentially expressed genes between two different groups were identified using the DESeq2 R package (version 1.46.0) with the criterion of *p* value less than 0.05. The volcano plot and heatmap were performed using the ggplot2 (version 4.0.0) and ComplexHeatmap (version 2.22.0) R package, respectively. GO analysis and KEGG analysis were performed using clusterProfiler (version 4.10.0) R package. The terms with *p* value < 0.05 were selected. COG annotation were performed by using COGclassifier (version 2.0.0).

### 4.11 Immunoblotting

Tissues were homogenized in RIPA buffer (Beyotime, P0013B) with protease inhibitors (Roche, 04693132001) and phosphatase inhibitors (Roche, 04906845001). The protein concentration was determined using a Pierce BCA Protein Assay Kit (Thermo, 23227) to guarantee equal protein quantity of each sample. The protein extracts were boiled for 10 min before separated by 10% sodium dodecyl sulfate‐polyacrylamide gel electrophoresis (SDS‐PAGE) gel and transferred onto PVDF membranes (Millipore, ISEQ00011). Afterward, membranes were blocked in 5% skimmed milk for 1 h at room temperature and then incubated with the indicated primary antibodies overnight at 4°C, followed by the corresponding secondary antibodies at room temperature for 1 h. The membranes were washed in 1×TBS with 0.1% Tween 20 and incubated with horseradish peroxidase (HRP)‐conjugated secondary antibody. β‐ACTIN, LAMINB1, GAPDH served as loading controls. Bands were visualized using an enhanced chemiluminescence (ECL) kit (Thermo, 32106) and detected by the Amersham Imager 680 System (Chemiluminescence). Grayscale values of bands were quantified using ImageJ software.

### 4.12 Cell Culture and Treatment

293T and Ishikawa cells were grown in high‐glucose Dulbecco's modified Eagle medium (DMEM) supplemented with 10% fetal bovine serum (FBS) and human endometrial stromal cells (hESC) were cultured in phenol red‐free DMEM/F12 media supplemented with 10% charcoal‐stripped FBS (vol/vol), 1×Penicillin‐Streptomycin at 37°C under 5% CO_2_. Cells were transiently transfected using Lipofectamine 3000 (Invitrogen, L3000015) according to the manufacturer's protocol. Co‐IP or Kinase‐IP Assay were performed at 48 h post‐transfection. hESC cells were treated with 1 µm Okadaic acid (Cayman, 10011490) for 30 min prior to harvest.

### 4.13 Immunoprecipitation

Cells were lysed with IP buffer (50 mm Hepes‐NaOH at pH 8.0, 150 mm NaCl, 2.5 mm ethylene glycol‐bis (β‐amino‐ethyl ether)‐N,N,N0,N0‐tetraacetic acid [EGTA], 1 mM dithiothreitol [DTT], 0.1% Tween20, and 10% glycerol), containing phosphatase and protease inhibitors. Cells protein lysates obtained by centrifugation were incubated with 1 µg anti‐FLAG or anti‐MYC antibody and 20 µL protein A/G beads (Santa Cruz, B2422) overnight at 4°C. The immunocomplexes were then washed four times using IP lysis buffer and separated by SDS‐PAGE gel. Target proteins were detected by standard Western blot. Antibodies used for IP for FLAG and MYC are the same as the ones used in WB.

### 4.14 Whole‐Mount Immunostaining for 3D Imaging

Samples were fixed in Dent's Fixative (Methanol: DMSO = 4:1) overnight in −20°C and then washed with 100% methanol for 3 times with 1 h each. The samples were then bleached with 3% H_2_O_2_ in methanol at 4°C overnight to eliminate pigmentation. After being washed in PBST (1% Triton) for 6 times with 1 h each, samples were incubated with anti‐CDH1 antibody (1:100) at 4°C in a rotor for 1 week. After incubation, the samples were washed by PBST (1% Triton) six times for 1 h each and incubated with 594‐conjugated Donkey Anti‐Rabbit lgG (H&L) (1:100, Proteintech, SA00013‐8) on a rotor for 4 days in a lightproof tube at 4°C. After six washes in PBST (1% Triton) at room temperature, the samples were protected from light until tissue clearing.

### 4.15 Tissue Clearing

A modified clear, unobstructed brain/body imaging cocktails and computational analysis (CUBIC) method was applied for tissue clearing. Briefly, the stained samples were gradually dehydrated into 100% methanol for storage in −20°C. When ready for analysis (day 1), samples were gradually rehydrated into PBS and then incubated in a 1: 1 solution of CUBIC Reagent 1 (CUBIC R1) and dH_2_O for 6 h at 37°C. Samples were then transferred into 100% CUBIC R1 at 37°C for 3 days. On day 4, following 3 washes in PBST (1% Triton) at room temperature, samples were transferred into a 1: 1 solution of CUBIC reagent 2 (CUBIC R2) and PBS for 6 h to overnight at 37°C, and then transferred into 100% CUBIC R2 until they appeared sufficiently clear.

### 4.16 3D Imaging and Processing

3D image acquisitions were performed by using a light sheet (ZEISS Z.1). Samples were immersed in CUBIC R2 for lightsheet confocal imaging using Lightsheet Z.1 5× detection optics. All files generated by ZEN were imported into Imaris (version 8.3, Bitplane) for visualization and 3D reconstruction. To obtain the 3D structure of the tissue, the surface tool was utilized for 3D rendering visualizations. To isolate a specific region of the tissue, the surface tool was manually used to segment the images and the mask option was selected for subsequent pseudo‐coloring. 3D images were generated using the “3D View” tools with magenta representing the luminal epithelium and blue representing the glandular epithelium.

### 4.17 Three‐Dimensional Glandular Quantification in Uterine Epithelial Architectures

The quantification of glandular units was performed in 3D‐reconstructed uterine epithelial structures. Gland segmentation was performed through differential demarcation of glandular epithelia from the luminal compartment utilizing the Imaris image analysis software. Individual glands were identified as contiguous glandular epithelial cell clusters maintaining complete structural connectivity and a singular basolateral connection point to the uterine lumen. Quantitative analysis was conducted through systematic sampling using orthogonal slice planes aligned along the XZ‐axis orientation. A standardized longitudinal uterine segment (500 µm in craniocaudal dimension) was selected for comprehensive enumeration, with all discernible glandular structures within this defined parametric volume being included in the final quantification.

### 4.18 Measurement of Serum P_4_ and E_2_ Levels

Mouse blood samples were collected on day 4 of pregnancy. P_4_ and E_2_ levels were measured by an enzyme‐linked immunosorbent assay kit (Cayman, 582601 and 501890, respectively).

### 4.19 Kinase‐IP Assay

Ishikawa cells were transfected with FKBP52‐FLAG and FKBP52(S224A)‐FLAG. Cells were lysed with IP buffer (50 mm Tris‐HCl, 150 mM NaCl, 1% Triton X‐100, pH 7.5) containing inhibitors of phosphatases and proteases. Ishikawa cell lysates (1 mg) were subjected to Kinase‐IP assays in a total of 200 µL IP buffer. Protein lysates were treated with 1 µm Okadaic acid at different time points. Anti‐phosphoserine antibodies (Abcam, ab9332) were used to pull down phosphorylated proteins. WB was employed to detect phosphorylated FKBP52 bands.

### 4.20 Subcellular Fractionation

Cytosolic and nuclear proteins were extracted using the Nuclear and Cytoplasmic Protein Extraction Kit (Beyotime, P0028). Cytosolic and nuclear samples from mice or cells for western blot analysis were prepared according to the manufacturer's protocol. LAMINB1 and GAPDH were used as nuclear and cytosolic loading controls, respectively.

### 4.21 *SAV1* Knockdown in hESC and Ishikawa Cells

*shRNA* of *SAV1* was constructed to pLKO.1‐Neo vector (Miaoling Biology, P33186) for hESC and Ishikawa cells. The *shRNA* sequences were designed from the website (https://rnaidesigner. thermofisher.com/rnaiexpress/design.do). *shSAV1*: CCATGATCTCTTCCAAAGAAT. For the virus infection, the expression vectors were transfected together with packaging plasmids (psPAX2 and pMD2.G) into HEK‐293T cells by lipofectamine 3000. After 48 h, the supernatants of the medium were collected and filtered with 0.45 µm mesh. The supernatant containing virus was stored in −20°C for cell infection. Cells were cultured in fresh media and subsequently infected with lentivirus overnight together with polybrene (Solarbio, P8761). Stably transfected hESC and Ishikawa cells were selected using G418 (Macklin, G6021). The knockdown efficiency was identified by western blotting.

### 4.22 In Situ Hybridization

Frozen uterine sections of each genotype were processed onto the same slide and hybridized with a digoxigenin (DIG)‐labeled probe to observe the signal under the bright field of a panoramic scanner (OLYMPUS, VS120). *Sav1, Msx1, Ltf, Prl3c1, Prl8a2, Bmp2, Wnt4* probes were generated according to SP6/T7 Transcription Kit (Roche, 11175025910) and the linear template DNA was transcribed into digoxin‐labeled RNA by DIG‐RNA Labeling Mix (Roche, 11277073910). After RNA transcription, 1 µL of DNaseI (Biolabs, M0303S) was added to digest the DNA template. Then, 2 µL of 0.5 M EDTA, 2.5 µL of 4 M LiCl, and 75 µL of cold ethanol were used to precipitate RNA overnight at −80°C. Subsequently, RNA probes were purified, and the working concentration was generally 1 µg/mL. The frozen sections were fixed in 4% PFAT (PFA+0.1% Tween 20 in PBS) at room temperature for 10 min, dehydrated and rehydrated with gradient methanol, followed by proteinase K (5 µg/mL) digestion and acetylation. Anti‐DIG‐AP (Roche, 11093274910) was applied to hybrid slides sealed with blocking solution. The color reaction was observed by BCIP/NBT alkaline phosphatase color development kit (Roche,11697471001). The primers of relative probes are listed in *SI Appendix*, Table S8.

### 4.23 RNAscope

RNAscope was performed using the RNAscope 2.5 HD Reagent Kit‐RED (ACD) according to the manufacturer's protocol. Probes against Mm‐*Fst* (Cat. No. 454331) and Mm‐*Hdc* (Cat. No. 490471) were obtained from ACD, and signals were visualized as red chromogenic deposits under bright‐field microscopy.

### 4.24 GST‐Pull Down and Protein Identification by Liquid Chromatography‐tandem mass spectrometry (LC‐MS/MS) Analysis

GST‐SAV1 construct was generated by subcloning the full‐length SAV1 into the pGEX‐6p‐1‐GST vector. GST‐SAV1 fusion protein and pGEX‐6p‐1‐GST vector were both expressed in *E. coli* BL21 (DE3) and purified using GST‐Sefinose Resin 4FF (Sangon Biotech, C600031) at 4°C. Uterine protein of wild‐type mice were harvested in lysis buffer (10 mM Hepes, 142.5 mM KCl, 5 mM MgCl_2_, 1 mM EGTA, 0.5% NP‐40) and incubated respectively with pGEX‐6p‐1‐GST and GST‐SAV1 GST‐Sefinose Resin 4FF overnight at 4°C. The immunoprecipitants were then washed by lysis buffer for 3 times and boiled for 10 min to elute bound proteins. Gel with target proteins were cut after standard western blot. For in‐gel trypsin digestion, gel slices were destained in 50 mM NH_4_HCO_3_/50% acetonitrile, dehydrated with acetonitrile (ACN), then reduced with DTT at 56°C for 1 h and alkylated with 55 mm iodoacetamide in the dark for 45 min. After washing with NH_4_HCO_3_ and dehydrating with ACN, gels were rehydrated with 10 ng/µL trypsin on ice for 1 h, followed by overnight digestion at 37°C. Peptides were extracted with 50% ACN/5% formic acid and 100% ACN, dried, and reconstituted in 2% ACN/0.1% formic acid. Peptides were separated on a homemade reversed‐phase column (15 cm × 75 µm) using a 27‐min gradient from 6% to 80% solvent B (0.1% formic acid in 98% ACN) at 400 nL/min on an EASY‐nLC 1000 system. MS analysis was performed on a Q Exactive Plus with an NSI source at 2.0 kV. Full MS scans (m/z 350–1800) were acquired at 70,000 resolution, followed by MS/MS scans at 17 500 resolution using normalized collision energy (NCE) 28. A data‐dependent method with dynamic exclusion (15 s) was used. Automatic gain control (AGC) was set to 5×10^4^. Data were processed using Proteome Discoverer 1.3 with mass tolerances of 10 ppm (precursor) and 0.02 Da (fragment), considering carbamidomethylation (fixed) and oxidation (variable). High‐confidence peptides with ion scores > 20 were retained.

### 4.25 MS Analysis of Protein Phosphorylation Site

FLAG‐tagged FKBP52 was equally overexpressed in *shSAV1* and *shNC* Ishikawa cells. Forty‐eight hours’ post‐transfection, proteins were immunoprecipitated using an anti‐FLAG antibody. The enriched samples were separated by SDS‐PAGE and stained with Coomassie blue. The band corresponding to FKBP52‐FLAG was excised and subjected to targeted phosphoproteomic analysis by MS to identify phosphorylation sites present exclusively in *shNC* cells. MS analysis was performed by JingJie PTM Biolab (Hangzhou) Co. Inc. For in‐gel trypsin digestion, gel pieces were destained in 50 mM NH_4_HCO_3_/50% ACN, reduced with 10 mM DTT at 56°C for 1 h, alkylated with 55 mm iodoacetamide in the dark for 45 min, then digested with 10 ng/µl trypsin at 37°C overnight. Peptides were extracted with 50% ACN/5% formic acid and 100% ACN, dried, and reconstituted in 2% ACN/0.1% formic acid. Peptides were separated on a homemade reversed‐phase column (25 cm length, 100 µm i.d.) using a 30‐min gradient from 6% to 80% solvent B (0.1% formic acid in 90% ACN) at 450 nL/minion via an EASY‐nLC 1200 UPLC system. MS/MS was performed on a Q Exactive Plus equipped with an NSI source (2.0 kV). Full MS scans (m/z 350–1800) were acquired at 70,000 resolution, followed by MS/MS scans at 17,500 resolution (NCE 28) in a data‐dependent mode with dynamic exclusion (15 s). AGC was set at 5×10^4^. Data were processed using Proteome Discoverer search engine (v.2.4). Tandem mass spectra were searched against sequence of target protein. Trypsin/P was specified as cleavage enzyme allowing up to 2 missing cleavages. The mass tolerance for precursor ions was set as 10 ppm in first search and the mass tolerance for fragment ions was set as 0.02 Da. Carbamidomethyl on Cys was specified as a fixed modification, and acetylation on protein N‐terminal, oxidation on Met, phosphorylation (STY) was specified as variable modifications. Peptide scoring is required to be higher than 0, and peptide confidence is set to Low for identification results.

### 4.26 Luciferase Assay for PR

Control and *SAV1*‐knockdown stromal cells were seeded in 12‐well plates and transfected at 80% confluency using Lipofectamine 3000. Control cells received 1 µg of PRE‐pGL3 basic and 20 ng of pRL‐TK per well. In addition, control cells were co‐transfected with 750 ng PRE‐pGL3 basic, 250 ng of either FKBP52‐FLAG or FKBP52(S224A)‐FLAG, and 20 ng pRL‐TK per well. After 6 h, the transfection medium was replaced with high‐glucose DMEM supplemented with 10% FBS, and cells were incubated for an additional 18 h. Prior to harvesting, cells were treated with 1 µm okadaic acid (Cayman Chemical, #10011490) for 30 min. Luciferase activity was measured using a commercial assay kit (Vazyme, #DD1205‐01) according to the manufacturer's instructions.

### 4.27 Modeling the Complex of MST1(MST2)/SAV1/FKBP52

Using AlphaFold2 Multimer software, the MST1(MST2)/SAV1 heterodimer was modeled with reference to the crystal structure model of MST2/SAV1 (PDB ID 6AO5), where the N‐lobe conformation of the MST1(MST2) Kinase domain was strictly modeled consistently with the conformation of the mouse cAMP‐dependent protein kinase in complex with ATP (PDB ID 1ATP). The C‐lobe of the kinase domain was modified according to the double‐phosphorylated MST1 crystal structure (PDB ID 3COM) or the double‐phosphorylated MST2 crystal structure (PDB ID 5DH3) in an open conformation, respectively. The substrate peptide of FKBP52 was modeled according to the crystal structure of the classical Ser/Thr kinase with a 5‐amino acid substrate (PDB ID 4JDJ).

### 4.28 Statistical Analysis

Statistical analyses were conducted using GraphPad Prism software (version 8.3). For comparisons between two groups, a two‐tailed Student's *t*‐test was applied when variances were assumed equal, whereas Welch's *t*‐test was used for groups with unequal variances. For comparisons involving a single independent variable, group means were compared using one‐way ANOVA followed by Tukey's multiple‐comparisons test. For comparisons involving two independent variables, two‐way ANOVA was used, followed by Dunnett's post hoc test. Normally distributed data are expressed as mean ± SEM. *p* value < 0.05 was considered statistically significant.

## Author Contributions

Y.H.S, Y.F.L, Z.Y.J, J.X.R, Z.Y.G and H.Z performed the experiments and analyzed the data; Y.H.S, X.Y.L and Y.F.L developed the conditional mouse model; Y.H.S, Y.F.L, Z.Y.J, W.W and J.Y designed experiments; Z.J.C, J.H.Y, W.W and J.Y supervised the study; Y.H.S, Z.Y.J, W.W and J.Y wrote the manuscript.

## Funding

This work was supported by the grant from the Natural Key Research and Development Program of China (2024YFC2706700, 2024YFC2702400), National Natural Science Foundation of China (32270906, 32171207, 32571436, 82471701), Natural Science Foundation of Shandong Province (ZR2024MC199) and Taishan Scholars Program for Young Experts of Shandong Province (tsqn202103023, tsqn202408004).

## Ethics Statement

All mice used in this study were housed in barrier facilities at Shandong University's Animal Care Facility according to the National Institutes of Health Guide for the Care and Use of Laboratory Animals, with the approval of the Institutional Animal Care and Use Committee of Shandong University (Permit No. SDU‐IACUC‐25066).

## Conflicts of Interest

The authors declare no conflicts of interest.

## Supporting information




**Supporting File**: advs77761‐sup‐0001‐SuppMat.docx.

## Data Availability

The raw sequence data reported in this paper have been deposited in the Genome Sequence Archive [55] in National Genomics Data Center [56]. China National Center for Bioinformation / Bejing Institute of Genomics, Chinese Academy of Sciences (GSA: CRA042700) that are publicly accessible at https://ngdc.cncb.ac.cn/gsa/s/2N8sY44j.
