# Supplementary material for: SAV1 in Cooperation With FKBP52 Participates in Implantation
Source: Adv Sci (Weinh). 2026 Sep 16:e77761. Online ahead of print. doi: 10.1002/advs.77761 (PMC13583225; doi:10.1002/advs.77761)
Supplement: Supplementary file 1 [file ADVS-9999-e77761-s001.docx]

Supplemental Figures, Tables and Figure legends

Figure S1. *Sav1^f/f^* and *Sav1^f/f^Pgr^Cre/+^* mice have comparable ovarian steroid hormone levels and expression patterns of uterine ESR1 and PGR. (A) High-magnification insets of *Sav1* in situ hybridization in uteri from day 1-4 and implantation sites from day 5-6 of pregnancy (Figure 1B). Scale bars, 20 μm. M, mesometrial pole; AM, antimesometrial pole. le, luminal epithelium; st, stroma; dec, decidua. Asterisks indicate the location of embryos. (B, C) Serum levels of progesterone (n = 5, B) and 17β-estradiol (n = 6, C) in *Sav1^f/f^* and *Sav1^f/f^Pgr^Cre/+^* mice on day 4 of pregnancy. (D) IHC staining of 3β-HSD and P450scc in *Sav1^f/f^* and *Sav1^f/f^Pgr^Cre/+^* day 4 ovaries. CL, corpus luteum; F, follicle. Scale bars, 200 μm. (E, F) IF of ESR1 (E) and PGR (F) co-stained with CK8 in *Sav1^f/f^* and *Sav1^f/f^Pgr^Cre/+^* uteri on day 4 of pregnancy. Scale bars, 200 μm. M, mesometrial pole; AM, antimesometrial pole. Statistical analysis was performed using two-tailed Student’s *t*-test. Data are presented as mean ± SEM. ns, not significant (*p* > 0.05).

Figure S2. Recombinant mLIF administration on day 5 of pregnancy partially rescued decidualization of *Sav1^f/f^Pgr^Cre/+^* mice. (A) 3D imaging of day 6 implantation sites from *Sav1^f/f^* (day 6) and *Sav1^f/f^Pgr^Cre/+^* (day 7, following mLIF on day 5) females. Luminal epithelium is shown in magenta, and glandular epithelium in blue. 3D images show glandless and compromised implantation chamber in *Sav1^f/f^Pgr^Cre/+^* mice. Scale bars, 300 μm. (B) IF of Cleaved-Caspase 3 and CK8 in day 6 implantation sites of *Sav1^f/f^* and day 7 implantation sites of *Sav1^f/f^Pgr^Cre/+^* uteri after mLIF induction on day 5. Asterisks indicate the location of embryos. Scale bars, 200 μm. (C) Representative photographs of uteri from *Sav1^f/f^* females on day 8 of pregnancy, and from *Sav1^f/f^Pgr^Cre/+^* females on day 8 and day 9 of pregnancy, following mLIF treatment on day 4 and day 5, respectively. (D, E) In situ hybridization of *Bmp2* (D)*, Prl3c1* and *Prl8a2* (E) in implantation sites of *Sav1^f/f^* (day 8) and *Sav1^f/f^Pgr^Cre/+^* (day 9, following mLIF on day 5) uteri. Arrowheads indicate the location of embryos. The dashed lines show the uterine vertical and horizontal axes. Scale bars, 500 μm. M, mesometrial pole; AM, antimesometrial pole.

Figure S3. Ablation of *Sav1* in the uterine epithelium has no effect on fertility. (A) Quantitative real-time PCR analysis of *Sav1* from *Sav1^f/f^* and *Sav1^f/f^Ltf^Cre/+^* mice respectively in luminal epithelium and stroma on day 4 of pregnancy (n = 5 mice per group) demonstrates an efficient and specific knockout of *Sav1* in the uterine epithelium. (B) Litter size in *Sav1^f/f^* (n = 20) and *Sav1^f/f^Ltf^Cre/+^* (n = 23) females. n, number of females examined. (C) IF of FOXA2 and CK8 in *Sav1^f/f^* and *Sav1^f/f^Ltf^Cre/+^* uteri on day 4 of pregnancy. Scale bars, 200 μm. (D) Day 5 implantation sites (blue bands) in *Sav1^f/f^* and *Sav1^f/f^Ltf^Cre/+^* females. (E) IF of COX2 and CK8 in day 5 implantation sites of *Sav1^f/f^* and *Sav1^f/f^Ltf^Cre/+^* uteri. Arrowheads indicate location of embryos. Scale bars, 200 μm. (F) Day 6 implantation sites (blue bands) in *Sav1^f/f^* and *Sav1^f/f^Ltf^Cre/+^* females. (G) In situ hybridization of *Sav1* mRNA in day 6 implantation sites of *Sav1^f/f^* and *Sav1^f/f^Ltf^Cre/+^* uteri. Asterisks indicate the location of embryos. Scale bars, 200 μm. (H) IF of FLK1 and ZO1 in day 6 implantation sites of *Sav1^f/f^* and *Sav1^f/f^Ltf^Cre/+^* mice. White dashed lines indicate primary decidual zones. Scale bars, 500 and 200 μm. (I) 3D imaging of day 6 implantation sites from *Sav1^f/f^* and *Sav1^f/f^Ltf^Cre/+^* females. Magenta denotes the luminal epithelium, whereas blue denotes the glandular epithelium. Images of CDH1 immunostaining and 3D rendered images show implantation chamber with normal glands in *Sav1^f/f^Ltf^Cre/+^* mice. Asterisks indicate the location of embryos. Scale bars, 300 μm. M, mesometrial pole; AM, antimesometrial pole. Statistical analysis was performed using two-tailed Student’s *t*-test. Data are presented as mean ± SEM. ns, not significant (*p* > 0.05), *****p* < 0.0001.

Figure S4. Transcriptomic alterations in uterine stromal cells upon *Sav1* deletion on day 4 and day 5 of pregnancy. (A) Volcano plot depicting differentially expressed genes in stromal cells of *Sav1^f/f^* and *Sav1^f/f^Pgr^Cre/+^* uteri on day 4 of pregnancy. Color bar represents the log_2_ fold change, and dot size represents the -log_10_ *p* value. (B) Enrichment analysis of the upregulated and downregulated genes in stromal cells upon SAV1 loss on day 4 pregnancy. GO enrichment (left), KEGG enrichment (right). *p* values were calculated using a hypergeometric test in clusterProfiler analysis without multiple testing correction. Bar height indicates the -log_10_ enrichment *p* value. (C) Volcano plot showing fold changes of differentially expressed genes in *Sav1^f/f^Pgr^Cre/+^* day 5 stroma versus *Sav1^f/f^* day 4 stroma. *p* values were determined by Negative Binomial Distribution. (D) GO functional analysis (left) and KEGG enrichment (right) of the upregulated and downregulated genes in stromal cells of *Sav1^f/f^Pgr^Cre/+^* day 5 stroma compared to *Sav1^f/f^* day 4 stroma. (E) Volcano plot showing fold changes of differentially expressed genes in *Sav1^f/f^Pgr^Cre/+^* day 5 versus *Sav1^f/f^Pgr^Cre/+^* day 4 stroma. *p* values were calculated by Negative Binomial Distribution. (F) GO functional analysis (left) and KEGG enrichment (right) of the upregulated and downregulated genes in stromal cells of *Sav1^f/f^Pgr^Cre/+^* day 5 stroma compared to *Sav1^f/f^Pgr^Cre/+^* day 4 stroma. (B, D and F) Purple and pink represent genes highly or lowly expressed in day 4 or day 5 *Sav1^f/f^Pgr^Cre/+^* uterus stromal cells, respectively.

**Figure S5. Transcriptomic analysis and validation of YAP/TAZ target genes in stromal and luminal epithelial cells of *Sav1^f/f^* and *Sav1^f/f^Pgr^Cre/+^* uteri on day 4 of pregnancy.** (A, B) Heatmaps showing the expression levels of YAP/TAZ target genes in luminal epithelial cells (LE) and stromal cells (ST) isolated from *Sav1^f/f^* and *Sav1^f/f^Pgr^Cre/+^* uteri on day 4 of pregnancy. Each column represents an individual biological replicate (n = 3 mice per group). Relative expression levels are indicated by the color scale. (C) Quantitative real-time PCR validation of selected YAP/TAZ target genes, including *Fos*, *Myc*, *Ccnd1*, *Vegfa*, and *Areg*, in day 4 pregnant uteri from *Sav1^f/f^* and *Sav1^f/f^Pgr^Cre/+^* mice. Gene expression was normalized to *Rpl7*. Each dot represents an individual mouse (n = 6 mice per group). Statistical analysis was performed using Welch’s *t*-test. Data are presented as mean ± SEM. ns, not significant (*p* > 0.05).

Figure S6. SAV1 regulates uterine gland development independently of the canonical Hippo pathway. (A) IF of FOXA2 and CK8 in *Sav1^f/f^* and *Sav1^f/f^Pgr^Cre/+^* uteri on PND7 and PND14. Scale bars, 100 μm. (B) Western blotting of main and active components of Hippo signaling in *Sav1^f/f^* and *Sav1^f/f^Pgr^Cre/+^* uteri on PND7 and PND14. GAPDH was used as a loading control. (C, D) IHC analysis of YAP (C) and pYAP (D) on PND7 and PND14 uteri from *Sav1^f/f^* and *Sav1^f/f^Pgr^Cre/+^* females. M, mesometrial pole; AM, antimesometrial pole. Scale bars, 100 μm. (E, F) Quantitative real-time PCR analysis of upstream core components and key downstream effectors of Hippo signaling in *Sav1^f/f^* and *Sav1^f/f^Pgr^Cre/+^* uterine tissues on PND7 (E) and PND14 (F). Each dot represents an individual mouse (n = 6 mice per group). Statistical analysis was performed using two-tailed Student’s *t*-test. Data are presented as mean ± SEM. ns, not significant (*p* > 0.05), **p* < 0.05, ***p* < 0.01.

**Figure S7.** **Temporal expression patterns of uterine receptivity-related genes in *Sav1^f/f^*** **and *Sav1^f/f^Pgr^Cre/+^* uteri during the peri-implantation period.** (A) Representative in situ hybridization analysis of *Ltf* expression in uterine sections from *Sav1^f/f^* and *Sav1^f/f^Pgr^Cre/+^* mice at 9:00 and 19:00 on day 3, at 9:00 on day 4, and additionally at 19:00 on day 5 of pregnancy for *Sav1^f/f^Pgr^Cre/+^* mice. (B) Representative immunohistochemical staining of IHH in uterine sections from *Sav1^f/f^* and *Sav1^f/f^Pgr^Cre/+^* mice at 9:00 and 19:00 on day 3, at 9:00 on day 4, and additionally at 19:00 on day 5 of pregnancy for *Sav1^f/f^Pgr^Cre/+^* mice. (C) Representative immunofluorescence staining of CTNNB1 (green) and CDH1 (red) in uterine sections from *Sav1^f/f^* and *Sav1^f/f^Pgr^Cre/+^* mice at 9:00 and 19:00 on day 3, at 9:00 on day 4, and additionally at 19:00 on day 5 of pregnancy for *Sav1^f/f^Pgr^Cre/+^* mice. Scale bars, 200 μm. M, mesometrial pole; AM, antimesometrial pole.

Figure S8. Knockdown of *SAV1* in hESCs resulted in reduced nuclear localization of FKBP52 and Hsp90. (A, B) Western blotting (A) and its quantitative graph (B) of *shNC* and *shSAV1* hESCs show efficient ablation of SAV1 (n = 3). β-ACTIN was used as a loading control. (C, D) Western blotting (C) and its quantitative graph (D) of FKBP52 and Hsp90 in *shNC* and *shSAV1* hESC whole-cell lysates (n = 3). β-ACTIN was used as a loading control. (E, F) Western blotting (E) and its quantitative graph (F) of nuclear FKBP52 and Hsp90 in *shNC* and *shSAV1* hESC nuclear extracts (n = 3). LAMINB1 and GAPDH were used as nuclear and cytoplasmic loading controls, respectively. (G, H) Western blotting (G) and its quantitative graph (H) of cytoplasmic FKBP52 and Hsp90 in *shNC* and *shSAV1* hESC cytoplasmic extracts (n = 3). LAMINB1 and GAPDH were used as nuclear and cytoplasmic loading controls, respectively. Statistical analysis was performed using two-tailed Student’s *t*-test. Data are presented as mean ± SEM. ns, not significant (*p* > 0.05), **p* < 0.05, ****p* < 0.001.

Figure S9. MST phosphorylates FKBP52 at serine 224. (A) Immunoblot analysis of MST2-MYC co-IP with FKBP52-FLAG from lysates of 293T cells transfected with the indicated plasmids. (B) Interaction between MST2-MYC and FKBP52-FLAG is enhanced with overexpression of SAV1-MYC. (C) Workflow for the mass spectrometric identification of FKBP52 phosphorylation sites in *shNC* and *shSAV1* Ishikawa cells overexpressed with FKBP52-FLAG (Created with BioRender.com). (D) Proteins immunoprecipitated with anti-FLAG antibody from *shNC* and *shSAV1* Ishikawa cells with OKA treatment were separated by SDS-PAGE and visualized by Coomassie Blue staining. The black boxes denote the overexpressed FLAG-tagged FKBP52 protein. (E) Mass spectrometric analysis revealed that serine 224 of FKBP52 is phosphorylated upon MST1 activation by okadaic acid. (F) The model of MST2/SAV1 heterodimer complexed with ATP and the substrate FKBP52. MST2 (light blue) and SAV1 (green) form heterodimer depending on SARAH coiled-coil domains. The peptide “LKPSYA” of FKBP52 (magenta) docks into the active site of MST2 kinase domain. The key phosphorylated residue T180 (blue) on the MST2 activation loop induces an open conformation to accommodate the substrate. The detailed interaction was illustrated in the dashed box on the right panel.

Figure S10. Delayed implantation and reactivation improved decidualization in *Sav1^f/f^Pgr^Cre/+^* mice. (A) Quantitative real-time PCR analysis of *Sav1* expression in uteri from *Sav1^f/f^*, *Sav1^f/f^Pgr^Cre/+^*, and ovariectomized P_4_-treated delayed *Sav1^f/f^Pgr^Cre/+^* females on day 4 of pregnancy. Gene expression was normalized to *Rpl7*. Each dot represents an individual mouse. (B) Western blot analysis of SAV1 protein expression in uteri from *Sav1^f/f^* and *Sav1^f/f^Pgr^Cre/+^* females under normal and ovariectomized P_4_-treated delayed conditions. GAPDH was used as the loading control. (C) Quantitative real-time PCR analysis of the glandular marker genes *Foxa2*, *Ttr*, *Prss28*, and *Prss29* in *Sav1^f/f^*, *Sav1^f/f^Pgr^Cre/+^*, and ovariectomized P_4_-treated delayed *Sav1^f/f^Pgr^Cre/+^* uteri. (D) Quantitative real-time PCR analysis of uterine receptivity-related genes, including *Hoxa10*, *Hoxa11*, *Hdc*, *Klf5*, *Wnt7b*, *Coch and Ltf* in the indicated groups. Gene expression was normalized to *Rpl7*. Each dot represents an individual mouse (n = 6 biologically independent animals). (E) Day 11 implantation sites (blue bands) induced by E_2_/LIF after 5 days of dormancy in *Sav1^f/f^* and *Sav1^f/f^Pgr^Cre/+^* females. P_4_ was administered from the day of ovariectomy until one day prior to evaluation. (F) In situ hybridization of *Bmp2* in delayed day 11 implantation sites from *Sav1^f/f^* and *Sav1^f/f^Pgr^Cre/+^* uteri. Asterisks indicate the location of embryos. Scale bars, 500 μm. (G) 3D visualization of implantation sites from delayed day 11 *Sav1^f/f^* and *Sav1^f/f^Pgr^Cre/+^* uteri. Magenta denotes the luminal epithelium, whereas blue denotes the glandular epithelium. CDH1 immunostaining images, along with 3D-rendered and segmented reconstructions of delayed day 11 implantation sites, reveal the implantation chamber induced by E_2_/LIF after 5 days of dormancy in *Sav1^f/f^Pgr^Cre/+^* females. Scale bars, 300 μm. (H) Day 13 implantation sites (blue bands) induced by E_2_/LIF after 5 days of dormancy in *Sav1^f/f^* and *Sav1^f/f^Pgr^Cre/+^* females. P_4_ was administered from the day of ovariectomy until one day prior to evaluation. (I, J) In situ hybridization of *Prl8a2* (I) and *Prl3c1* (J) in delayed day 13 implantation sites of *Sav1^f/f^* and *Sav1^f/f^Pgr^Cre/+^* uteri. The dashed lines show the uterine vertical and horizontal axes. Arrowheads indicate the location of embryos. Scale bars, 500 μm. M, mesometrial pole; AM, antimesometrial pole. Statistical analysis was performed using one-way ANOVA followed by Tukey’s multiple-comparisons test. Data are presented as mean ± SEM. ns, not significant (*p* > 0.05), **p* < 0.05, ***p* < 0.01, *****p* < 0.0001.

**Figure S11. Reduced *SAV1* expression and diminished progesterone responsiveness in uterine stromal and decidual cells from patients with pregnancy loss.** (A) Violin plots showing the expression of *SAV1* and progesterone-responsive genes (*WNT4*, *HOXA10* and *HAND2*) in stromal cells from normal fertile female controls and patients with recurrent implantation failure (RIF). (B) Violin plots showing the expression of *SAV1* and progesterone-responsive genes in stromal cells from normal fertile female controls and patients with recurrent miscarriage (RM). (C) Violin plots showing the expression of *SAV1* and progesterone-responsive genes in stromal cells from normal fertile female controls and patients with recurrent spontaneous abortion (RSA). (D) Violin plots showing the expression of *SAV1* and progesterone-responsive genes in decidual cells from normal fertile female controls and patients with RSA. Statistical analysis was performed using Wilcoxon rank-sum test. ***p* < 0.01, *****p* < 0.0001.

Figure S12. Schematic summary of SAV1-mediated regulation of progesterone receptor activity during uterine receptivity. The MST-SAV1 complex modulates FKBP52 phosphorylation to fine-tune progesterone receptor (PR) activity. This regulation ensures the timely progression of the uterus from the transitional to the receptive phase under coordinated estrogen signaling, thereby facilitating successful implantation. Our study identifies stromal SAV1 as a pivotal regulator of uterine receptivity, acting through a noncanonical Hippo pathway mechanism to scaffold MST1/2-mediated phosphorylation of FKBP52 and thereby promote PR activity. Loss of SAV1 disrupts this axis, resulting in progesterone resistance, temporal misalignment of receptivity, and implantation failure.

Supplementary Tables

Table S1. Embryo implantation rates in *Sav1^f/f^Pgr^Cre/+^* mice following supplementation with mLIF on day 4 or 5.

|  |  |  | Sav1^f/f^Pgr^Cre/+^ |  |
| --- | --- | --- | --- | --- |
| Treatment | Day of Pregnancy | No. of mice | No. of mice with IS(%) | No. of ISs |
| D4 + mLIF | 5 | 8 | 12.5（1/8） | 0.9 ± 0.9 |
|  | 6 | 8 | 37.5（3/8） | 1.3 ± 0.7 |
|  | 8 | 9 | 55.6（5/9） | 2.4 ± 1.1 |
| D5 + mLIF | 6 | 8 | 100（8/8） | 7.0 ± 0.6 |
|  | 7 | 10 | 90（9/10） | 7.3 ± 0.9 |
|  | 9 | 8 | 100（8/8） | 7.8 ± 0.6 |

**Table S2.** Temporal effects of LIF supplementation on implantation in *Sav1^f/f^Pgr^Cre/+^* mice.

|  | Sav1^f/f^Pgr^Cre/+^ | | | |
| --- | --- | --- | --- | --- |
| Treatment | Day of Pregnancy | No. of mice | No. of mice with IS(%) | No. of ISs |
| D3 19:00 + mLIF | 5 | 8 | 0（0/8） | 0.0 ± 0.0 |
| D4 9:00 + E_2_ and mLIF | 5 | 8 | 0（0/8） | 0.0 ± 0.0 |

**Table S3.** Embryo implantation rates in *Sav1^f/f^Pgr^Cre/+^* mice following progesterone (P_4_) supplementation from Day 1 to Day 4 of pregnancy and additional mLIF on Day 4.

|  |  |  | Sav1^f/f^Pgr^Cre/+^ |  |
| --- | --- | --- | --- | --- |
| Treatment | Duration | No. of mice | No. of mice with IS(%) | No. of ISs |
| P_4_ | Days 1-4 | 9 | 55.6（5/9） | 2.3 ± 1.0 |
| mLIF | Day 4 |  |  |  |

Table S4. Embryo implantation rates in *Sav1^f/f^* and *Sav1^f/f^Pgr^Cre/+^* mice across different delayed durations.

| Treatment | Days after E_2_ | Sav1^f/f^ | | Sav1^f/f^Pgr^Cre/+^ | | *p* value |
| --- | --- | --- | --- | --- | --- | --- |
|  |  | No. of mice with IS(%) | No. of ISs | No. of mice with IS(%) | No. of ISs |  |
| Delayed Implantation | 1 | 100（8/8） | 5.3 ± 0.8 | 100（9/9） | 5.8 ± 0.7 | 0.61 |
|  | 2 | 100（8/8） | 5.3 ± 1.1 | 100（8/8） | 4.6 ± 0.8 | 0.64 |
|  | 4 | 100（8/8） | 6.6 ± 0.7 | 100（8/8） | 5.5 ± 1.0 | 0.38 |

Statistical analysis was performed using two-tailed Student’s *t*-test.

**Table S5.** Embryo implantation rate in *Sav1^f/f^Pgr^Cre/+^* mice after LIF reduction without E_2_.

| Treatment | Sav1^f/f^Pgr^Cre/+^ | |
| --- | --- | --- |
|  | No. of mice with IS(%) | No. of ISs |
| Delayed Implantation（one day after LIF) | 100（9/9） | 5.7 ± 0.7 |
|  |  |  |
|  |  |  |

Table S6. Antibodies used in this study.

| Antibodies | Company | Catalog | Application (Dilution) |
| --- | --- | --- | --- |
| E-cadherin | CST | #3195 | 3D Imaging (1/100)/IF (1/300) |
| Cleaved caspase 3 | CST | #9661 | IF (1/300) |
| β-catenin | CST | #8480 | IF (1/300) |
| FOXA2 | Seven Hills Bioreagents | WRAB-1200 | IF (1/300) |
| COX2 | Cayman chemical | 160106 | IF (1/300) |
| Cytokeratin 8 | Millipore | NP112447 | IF (1/1000) |
| FLAG | Sigma | F1804 | IP (1/500) / IB (1/5000) |
| Phospho-Histone H3 (Ser10) | CST | #9701 | IF (1/300) |
| Progesterone Receptor A/B | CST | #8757 | IF (1/300) |
| ZO-1 | Invitrogen | 2251979 | IF (1/300) |
| GAPDH | Santa cruz | sc-365062 | IB (1/3000) |
| MYC | Zenbio | 390003 | IP (1/250) / IB (1/1000) |
| Estrogen Receptor alpha | Zenbio | R24251 | IF (1/300) |
| VEGF Receptor 2 | CST | #2479 | IF (1/300) |
| SAV1 | Novus Biology | NBP2-13282 | IF (1/50) / IB (1/1000) |
| FKBP52 | Proteintech | 10655-1-AP | IHC (1/300) / IB(1/1000) |
| FKBP52 | Abcam | Ab129097 | IB(1/1000) |
| Ki67 | Invitrogen | 14-5698-82 | IF (1/1000) |
| HSP90 | CST | #4877 | IHC (1/300) / IB (1/1000) |
| LAMINB1 | Proteintech | 12987-1-AP | IB (1/1000) |
| β-ACTIN | Abmart | T40104 | IB (1/5000) |
| HIPPO Signaling Antibody Sampler Kit | CST | #8579 | IB (1/1000) |
| TEAD1 | CST | #12292T | IB (1/1000) |
| TEAD2 | Proteintech | 21159-1-AP | IB (1/1000) |
| TEAD3 | Proteintech | 13120-1-AP | IB (1/1000) |
| TEAD4 | Proteintech | 12418-1-AP | IB (1/1000) |
| YAP1 | Proteintech | 13584-1-AP | IHC (1/300)/ IB (1/1000) |
| TAZ | Immunoway | YN1701 | IB (1/1000) |
| Phospho-P38 | Huabio | HA722150 | IHC (1/300) |
| IHH | Proteintech | 13388-1-AP | IHC (1/300) |
| Phospho-Serine | Abcam | ab9332 | IP (1/250) |
| P450scc | Abcam | ab272494 | IHC (1/300) |
| 3β-HSD | Abcam | ab65156 | IHC (1/300) |
| HRP-conjugated Affinipure Donkey Anti-Mouse lgG ( H&L) | Proteintech | SA00001-8 | IB (1/5000) |
| HRP-conjugated Affinipure Donkey Anti-Rabbit lgG ( H&L) | Proteintech | SA00001-9 | IB (1/5000) |
| 488-conjugated Affinipure Donkey Anti-Rabbit lgG ( H&L) | Proteintech | SA00013-6 | IF (1/500) |
| 594-conjugated Donkey Anti-Rabbit lgG ( H&L) | Proteintech | SA00013-8 | IF (1/500) |
| Alexa fluor 594 Donkey Anti-rat lgG ( H&L) | Invitrogen | A21209 | IF (1/500) |
| Goat Anti-Mouse lgG HRP (light chain specific) | Abmart | M21004 | IB (1/2500) |

IP, immunoprecipitation; IB, immunoblot; IF, immunofluorescence; IHC, immunohistochemistry.

**Table S7.** RT-qPCR primers used in this study.

| Genes | Primers (5' → 3') |
| --- | --- |
| *mSav1-q*PCR*-F* | AGCAGATGTCCCTCGAGAGT |
| *mSav1-q*PCR*-R* | GACTCTGTGGCATCCTCTGG |
| *mLif-q*PCR*-F* | TCAACTGGCACAGCTCAATGGC |
| *mLif-q*PCR*-R* | GGAAGTCTGTCATGTTAGGCGC |
| *mFoxa2-qPCR-F* | TCCGACTGGAGCAGCTACTAC |
| *mFoxa2-qPCR-R* | GCGCCCACATAGGATGACA |
| *mTtr-qPCR-F* | GTCAAAGTCCTGGATGCT |
| *mTtr-qPCR-R* | GGGAAATGCCAAGTGTC |
| *mPrss28-qPCR-F* | TGACGACATGCTTTGTGCTGGC |
| *mPrss28-qPCR-R* | CCCACCTGTATCCACTTGTTGC |
| *mPrss29-qPCR-F* | GGTCACCAAGAAGGATGTGTGC |
| *mPrss29-qPCR-R* | CTTTACCTGCACCTGCTGTAGG |
| *mSpink1-qPCR-F* | CAGCTAAGGTGACTGGAAAAGAG |
| *mSpink1-qPCR-R* | AATGAGGACAGGCTCTATGCGTT |
| *mCxcl15-qPCR-F* | GGTGATATTCGAGACCATTTACTG |
| *mCxcl15-qPCR-R* | GCCAACAGTAGCCTTCACCCAT |
| *mWfdc3-qPCR-F* | CCTCTTCCATGTCAGGAGCTGT |
| *mWfdc3-qPCR-R* | CCAGGATTCTGGGACATTGACC |
| *mRpl7-qPCR-F* | AGCTGGCAACTTCTATGTGC |
| *mRpl7-qPCR-R* | CGCAGCATGTTAATTGAAGCC |
| *mStk4-qPCR-F* | AGCTTGCAGACTTTGGAGTCGC |
| *mStk4-qPCR-R* | GAGACCAGATGTCTGCCACACA |
| *mStk3-qPCR-F* | CCAAACGGCATGAAGAGCAGCA |
| *mStk3-qPCR-R* | CTCCTTCACTCATCGTGCTGGT |
| *mLats1-qPCR-F* | TGGGACAACCTCCTTTCTTGGC |
| *mLats1-qPCR-R* | TGAGGTCAGAGGCTTCAGGACT |
| *mLats2-qPCR-F* | GCACTGGATTCAGGTGGACTCA |
| *mLats2-qPCR-R* | CGACAGTTGGAAACATCGTCCC |
| *mMob1a-qPCR-F* | CACCAGCACTTTGACTCCGTGA |
| *mMob1a-qPCR-R* | TCCTGAAGTGGTGCCAACTCAC |
| *mYap1-qPCR-F* | CCAGACGACTTCCTCAACAGTG |
| *mYap1-qPCR-R* | GCATCTCCTTCCAGTGTGCCAA |
| *mWwtr1-qPCR-F* | GTCACCAACAGTAGCTCAGATCC |
| *mWwtr1-qPCR-R* | GTTGCTGAGGAAGTCTTCTGGAG |
| *mTead1-qPCR-F* | TCCGCTTTCCTTGAACAGCAGAG |
| *mTead1-qPCR-R* | GGGTCACTGTAAGAATGGTTGGC |
| *mTead2-qPCR-F* | AAGGTCTGCTCCTTTGGCAAGC |
| *mTead2-qPCR-R* | CTGACGGAGCTTGTGCAGGAAA |
| *mTead3-qPCR-F* | ATCGTCTCTGCCAGCGTTCTAC |
| *mTead3-qPCR-R* | CTGAGAAGGTCCAGGCTGTTGT |
| *mTead4-qPCR-F* | GCTCTGGATGTTGGAGTTCTCG |
| *mTead4-qPCR-R* | TTGGGCTTGACTGGCTGATGTG |
| *mCcn1-qPCR-F* | GTGAAGTGCGTCCTTGTGGACA |
| *mCcn1-qPCR-R* | CTTGACACTGGAGCATCCTGCA |
| *mCcn2-qPCR-F* | TGCGAAGCTGACCTGGAGGAAA |
| *mCcn2-qPCR-R* | CCGCAGAACTTAGCCCTGTATG |
| *mAxl-qPCR-F* | GGTGTTTGAGCCAACCGTGGAA |
| *mAxl-qPCR-R* | GCCACCTTATGCCGATCTACCA |
| *mAnkrd1-qPCR-F* | GCTTAGAAGGACACTTGGCGATC |
| *mAnkrd1-qPCR-R* | GACATCTGCGTTTCCTCCACGA |
| *mAmotl2-qPCR-F* | CAGAGGGACAATGAGCGATTGC |
| *mAmotl2-qPCR-R* | TCACGCTTGGAAGAGGTCCTCA |
| *mPrl8a2-qPCR-F* | TGTCACAAACCCACCAG |
| *mPrl8a2-qPCR-R* | CAATCTTGCCCAGTTAT |
| *mFos-qPCR-F* | GGGGACAGCCTTTCCTACT |
| *mFos-qPCR-R* | TGCGCAAAAGTCCTGTGTG |
| *mMyc-qPCR-F* | CAGTGGTCTTTCCCTACCCG |
| *mMyc-qPCR-R* | GGAGAGAAGGCCGTGGAATC |
| *mCcnd1-qPCR-F* | TGCTGCAAATGGAACTGCTT |
| *mCcnd1-qPCR-R* | GGTCTGCTTGTTCTCATCCG |
| *mVegfa-qPCR-F* | AGCAGAAGTCCCATGAAGTGAT |
| *mVegfa-qPCR-R* | CACACAGGACGGCTTGAAGA |
| *mAreg-qPCR-F* | GACAAGAAAATGGGACTGTGC |
| *mAreg-qPCR-R* | GGCTTGGCAATGATTCAACT |
| *mHoxa10-qPCR-F* | CTACGATGCTGCGGACAAA |
| *mHoxa10-qPCR-R* | CTGCGACAGGCGGAAGT |
| *mHoxa11-qPCR-F* | CGGTGGCTCCGGTGG |
| *mHoxa11-qPCR-R* | TTGACTTGACGGTCGGTGAG |
| *mHdc-qPCR-F* | TGGAGCCCTGTGAATACCGT |
| *mHdc-qPCR-R* | GCACATTTGGAGTCACCTGC |
| *mKlf5-qPCR-F* | CCGGAGACGATCTGAAACACG |
| *mKlf5-qPCR-R* | GTTGATGCTGTAAGGTATGCCT |
| *mWnt7b-qPCR-F* | CTTCACCTATGCCATCACGG |
| *mWnt7b-qPCR-R* | TGGTTGTAGTAGCCTTGCTTCT |
| *mCoch-qPCR-F* | TGCTGTCCATAGGGGAGTGAT |
| *mCoch-qPCR-R* | CCGAGGAGTAGTTCTCTCGAC |
| *mLtf-qPCR-F* | GGTGGCAGAACGAGATG |
| *mLtf-qPCR-R* | GGTCGCAGTTTGTAGGG |

**Table S8.** Probe primers of in situ hybridization used in this study.

| Genes | Primers (5' → 3') |
| --- | --- |
| *Sav1-probe-F* | AGTACGTGAAGAAGGAGACG |
| *Sav1-probe-R* | AGAACGTCTTGCCATGCTGC |
| *Bmp2-probe-F* | CGTCCTCAGCGAATTTGAGT |
| *Bmp2-probe-R* | TGCCACGATCCAGTCATTCCA |
| *Wnt4-probe-F* | ATGAGCCCCCGTTCGTGCCT |
| *Wnt4-probe-R* | ACCGGCACGTGTGCATCTCCA |
| *Prl8a2-probe-F* | TCAACCTCACTTCTGGGCAC |
| *Prl8a2-probe-R* | GAGCAGCCATTCTCTCCTGTT |
| *Prl3c1-probe-F* | ATGTTCCTGTGGGTATATGT |
| *Prl3c1-probe-R* | GCAAATGGTTTTGATTTTGCC |
| *Msx1-probe-F* | TGCTGCTATGACTTCTTTGC |
| *Msx1-probe-R* | CTAAGTCAGGTGGTACATGC |
| *Ltf-probe-F* | TTGTGTGAACAGACCAGTGGAA |
| *Ltf-probe-R* | TTCTACCTAAAGTGACAGCA |
